# The Arabidopsis AtUNC-93 Acts as a Positive Regulator of Abiotic Stress Tolerance and Plant Growth via Modulation of ABA Signaling and K^+^ Homeostasis

**DOI:** 10.3389/fpls.2018.00718

**Published:** 2018-05-30

**Authors:** Jianhua Xiang, Xiaoyun Zhou, Xianwen Zhang, Ailing Liu, Yanci Xiang, Mingli Yan, Yan Peng, Xinbo Chen

**Affiliations:** ^1^College of Bioscience and Biotechnology, Hunan Agricultural University, Changsha, China; ^2^Institute of Ecological Landscape Restoration, Hunan University of Science and Technology, Xiangtan, China; ^3^School of Life Sciences, Hunan University of Science and Technology, Xiangtan, China

**Keywords:** *Arabidopsis thaliana*, abiotic stress, plant growth, abscisic acid, K^+^ homeostasis, AtUNC-93

## Abstract

Potassium (K^+^) is one of the essential macronutrients required for plant growth and development, and the maintenance of cellular K^+^ homeostasis is important for plants to adapt to abiotic stresses and growth. However, the mechanism involved has not been understood clearly. In this study, we demonstrated that AtUNC-93 plays a crucial role in this process under the control of abscisic acid (ABA). AtUNC-93 was localized to the plasma membrane and mainly expressed in the vascular tissues in *Arabidopsis thaliana*. The *atunc-93* mutants showed typical K^+^-deficient symptoms under low-K^+^ conditions. The K^+^ contents of *atunc-93* mutants were significantly reduced in shoots but not in roots under either low-K^+^ or normal conditions compared with wild type plants, whereas the *AtUNC-93*-overexpressing lines still maintained relatively higher K^+^ contents in shoots under low-K^+^ conditions, suggesting that AtUNC-93 positively regulates K^+^ translocation from roots to shoots. The *atunc-93* plants exhibited dwarf phenotypes due to reduced cell expansion, while *AtUNC-93*-overexpressing plants had larger bodies because of increased cell expansion. After abiotic stress and ABA treatments, the *atunc-93* mutants was more sensitive to salt, drought, osmotic, heat stress and ABA than wild type plants, while the *AtUNC-93*-overexpressing lines showed enhanced tolerance to these stresses and insensitive phenotype to ABA. Furthermore, alterations in the *AtUNC-93* expression changed expression of many ABA-responsive and stress-related genes. Our findings reveal that AtUNC-93 functions as a positive regulator of abiotic stress tolerance and plant growth by maintaining K^+^ homeostasis through ABA signaling pathway in Arabidopsis.

## Introduction

Plants have developed a wide range of adaptive mechanisms to control growth and development under various abiotic stresses. The regulation of ion flux, including ion influx and efflux, in maintaining an appropriate level of ion homeostasis plays an essential role in improving plant adaptation to abiotic stresses, particularly salt stress. Salt injury to plants is mainly caused by Na^+^ toxicity. Plant cells depend on Na^+^ exclusion and sequestration to avoid Na^+^ toxicity (Osakabe et al., 2014). The SOS pathway is known to regulate plant salt stress tolerance by Na^+^ exclusion. The Na^+^/H^+^ antiporter SOS1, regulated by the SOS2-SOS3 complex, is involved in Na^+^ efflux from cytosol to the extracellular environment across the plasma membrane to maintain a low concentration of Na^+^ in cells (Qiu et al., 2002; Zhu, 2003). The CBL10-CIPK24 complex may predominantly function in Na^+^ sequestration into the vacuole in plant shoots (Kim et al., 2007; Quan et al., 2007). In addition, an increasing number of reports demonstrate that cytosolic potassium (K^+^) retention and maintenance of K^+^/Na^+^ homeostasis is important for plants to adapt to and tolerate salt stress. The endophytic fungus *Piriformospora indica* promotes Arabidopsis growth by maintaining K^+^/Na^+^ homeostasis under salt stress conditions (Abdelaziz et al., 2017). *Jatropha curcas* adapts to salt stress which is related to transport and selectivity of K^+^ and K^+^/Na^+^ homeostasis (Silva et al., 2015). *Arbuscular mycorrhizal* symbiosis alleviates salt stress in black locust through K^+^/Na^+^ homeostasis (Chen et al., 2017). Therefore, the maintenance of K^+^/Na^+^ homeostasis is one of the important mechanisms which plants use to increase their adaptation to abiotic stresses. K^+^ is not only essential for stress responses, but also functions as an important role in cell expansion regulated by turgor pressure. The K^+^ transport systems triple mutants, *kup268* and *kup68 gork*, exhibited enhanced cell expansion and significantly decreased drought tolerance in Arabidopsis, suggesting that these K^+^ transporters act as key factors in K^+^ homeostasis in both drought stress responses and cell growth (Osakabe et al., 2013). The Na^+^/H^+^ antiporters NHX5 and NHX6 play important roles in cellular K^+^/Na^+^ homeostasis as the *nhx56* double knockout mutant reduced Arabidopsis tolerance to salt tress and salinity stress and exhibited suppressed growth, with smaller and fewer cells (Bassil et al., 2011).

The phytohormone abscisic acid (ABA) plays a pivotal role in stress tolerance, and growth and development (Finkelstein et al., 2002; Weiner et al., 2010). In ABA signaling pathway, the PYR/PYL/RCAR receptor proteins can disrupt the interaction between the SnRK2s and PP2Cs in the presence of ABA, thus preventing the PP2Cs-mediated dephosphorylation of the SnRK2s and resulting in the activation of the SnRK2s. The relieved SnRK2s (SnRK2.2/2.3/2.6) can then phosphorylate ABFs to activate ABA-responsive genes (Ma et al., 2009; Park et al., 2009). Recent studies have reported that ABA signaling can control membrane transport systems in response to drought and salt stresses by maintaining ion homeostasis in plant (Osakabe et al., 2014). The K^+^ transporter KUP6 can be phosphorylated by SnRK2.6, a key component of ABA signaling, suggesting that KUP6 plays an important role in K^+^ homeostasis mediated by ABA signaling (Osakabe et al., 2013). An outward anion channel SLAC1 is directly activated by SnRK2.6 that is involved in stomatal closure regulated by ABA signaling (Lee et al., 2009). BdCIPK31, a calcineurin B-like protein-interacting protein kinase in *Brachypodium distachyon*, plays a positive role in regulating drought and salt stress tolerance by maintaining K^+^/Na^+^ homeostasis through ABA signaling pathway (Luo et al., 2017).

The UNC-93 in *Caenorhabditis elegans* was identified to be a component of a multi-subunit K^+^ channel complex that coordinates muscle contraction, and it may be a regulatory subunit of this channel (de la Cruz et al., 2003). But there is no functional report so far regarding UNC-93 domain protein in plants. In this study, we demonstrate that AtUNC-93, a novel UNC-93 domain protein, regulates K^+^ translocation from roots to shoots in *Arabidopsis thaliana*, and plays a critical role in abiotic stress tolerance and plant growth by maintaining K^+^ homeostasis through ABA-dependent signal transduction pathways.

## Materials and Methods

### Plant Materials and Growth Conditions

*Arabidopsis thaliana* ecotype Columbia was used in this study. The T-DNA insertion mutants, including *atunc-93-1* (CS879007) and *atunc-93-2* (SALK_010430C) (Columbia-0 background), were obtained from the Arabidopsis Biological Resource Center (ABRC). Homozygous individuals were isolated in the F2 progeny by PCR genotyping. The expression levels of the mutant genes were confirmed by RT-PCR. Homozygous mutants were used for experiments.

For non-sterile culture, Arabidopsis plants were grown in potting soil mixture (rich soil: vermiculite = 1:1, v/v) and kept in growth chambers at 22°C with illumination at 120 μmol m^-2^ s^-1^ for a 16 h daily light period. The relative humidity was approximate 65% (± 5%). For sterile culture, seeds were surface sterilized and placed on 1/2 Murashige and Skoog (MS) medium containing 3% (w/v) sucrose and 2.5g L^-1^ phytagel and kept in growth chambers as described above after 3 days of vernalization in darkness at 4°C.

### Vector Constructions and Arabidopsis Transformation

For generation of *AtUNC-93*-overexpressing lines, the construct was generated by cloning the coding sequence of *AtUNC-93* into the pCAMBIA1301-Multi (modified from pCAMBIA1301) vector under the control of the cauliflower mosaic virus 35S promoter (Xiang et al., 2013). The construct was transformed into wild type Arabidopsis. For generation of complementation lines of *atunc-93* mutants, the *35S:AtUNC-93* construct was transformed into *atunc-93-1* and *atunc-93-2*, respectively. Arabidopsis transformation with *Agrobacterium tumefaciens* strain GV3101 was performed by the floral-dip method (Clough and Bent, 1998). Analyses of transgenic lines were performed on homozygous T3 progeny plants.

### Growth Assays and Stress Treatments

To observe *atunc-93* mutants and transgenic plants growth phenotypes under normal conditions grown in soil, plant growth was monitored and photographed at the indicated times in figure legends. To investigate the stem cell size, the plant stems were sliced into the paraffin sections after 6 weeks growth in soil and observed by inverted microscopy. For measurement of hypocotyl and root length, the seedlings grown on 1/2 MS medium in the light for 7 days or darkness for 10 days in the vertical position were photographed. The hypocotyl and primary root lengths of seedlings were measured using a ruler. The dry weight of seedling roots and shoots was measured after 14 days of growth on 1/2 MS medium. Forty plants of each line were analyzed in independent experiments, and all experiments were repeated three times.

For NaCl or mannitol treatments, 4-day-old seedlings were transferred to 1/2 MS medium supplemented with different concentrations of NaCl or mannitol. Results were documented photographically after 14 or 20 days of treatment. For heat stress, 4-day-old seedlings grown on 1/2 MS medium plates were exposed to one of the following heat treatments: 45°C for 1 to 3 h; 38°C for 90 min, followed by 45°C for 2 to 4 h; or 38°C for 90 min, followed by 2 h of recovery at 22°C and then 2 to 4 h at 45°C. For cold stress, 4-day-old seedlings were incubated at 4°C for 10 days. After heat or cold treatments, the plates were returned to 22°C, and viability was assessed daily for 7 days. For drought stress, potted seedlings were grown under normal watering conditions for 14 days and then stressed by completely depriving them of irrigation for 16 days (normal drought stress) or 19 days (severe drought stress). The plants were rewatered when significant differences in wilting were observed and survival was assessed at 2 days after rewatering. For ABA treatments, seeds were germinated on the ABA-free medium after 48 h stratification and transferred to 1/2 MS medium supplemented with different concentrations (0, 1, 3, 5, and 10 μM) of ABA in the vertical position. Seedling growth was investigated at 7 days after transfer, and the length of primary roots was measured. Several independent experiments were performed in this study to obtain statistically significant data.

For water loss measurement, rosette leaves were detached from 6-week-old plants, weighed immediately, and then placed on the laboratory bench. Weight loss of the detached leaves was monitored at the designated time periods. Water loss rate was expressed as the percentage of initial fresh weight. Three leaves per plant were excised, and ten plants of each genotype and transgenic line were tested in independent experiments repeated at least three times.

### GUS Assays

To analyze the histological expression patterns of *AtUNC-93* in Arabidopsis, the 2100 bp fragment before the ATG codon of *AtUNC-93* was cloned from wild type Arabidopsis. The *AtUNC-93* promoter fragment was fused in front of the *GUS* coding sequence in pCAMBIA1305 vector. This construct was confirmed by sequencing and introduced into wild type Arabidopsis plants by using *A. tumefaciens*-mediated transformation. The T3 homozygous transgenic plants of *ProAtUNC-93:GUS* were stained by submersion in staining solution at 37°C until sufficient staining developed as described previously (Jefferson et al., 1987). Then the chlorophyll in green tissues was cleared by 75% (v/v) ethanol. Images were taken under a stereomicroscope.

### Subcellular Localization Analysis

For generation of AtUNC-93-GFP fusion protein, the coding sequence of *AtUNC-93* was fused with *GFP* in the pBI121-GFP vector. The construct confirmed by sequencing and empty vector were transformed into *A. tumefaciens* strain GV3101 and then transiently transferred to onion (*Allium cepa*) epidermal cells mediated by *A. tumefaciens*. The fluorescence of GFP in the transformed onion epidermal cells was imaged using a confocal laser scanning microscope (Leica sp5) after the onion epidermal cells were incubated at 25°C for 16 h.

### Real-Time PCR and RT-PCR

To check the expression level of *AtUNC-93* gene under abiotic stress and ABA treatments, 14-day-old Arabidopsis plants were transferred from normal 1/2 MS medium to medium containing 150 mM NaCl, 200 mM mannitol or 3 μM ABA, respectively. For heat and cold stress, 14-day-old seedlings were transferred to a growth chamber at 38 or 4°C. Samples were taken after treatment for 0, 1, 5, 10, and 24 h separately. To detect the transcript levels of *AtUNC-93* in different tissues, tissue samples of roots, stems, rosette leaves, stem leaves, flowers, and siliques were collected from 6-week-old Arabidopsis plants. Seedlings were obtained from 14-day-old seedlings grown on normal 1/2 MS medium. To test the expression levels of stress-related and ABA-responsive genes, 14-day-old seedlings were treated with NaCl, mannitol or ABA as described above for 5 h. For drought stress, 14-day-old seedlings grown in soil spots were exposed to water deficit stress by not watering them for 2 weeks. Relative expression of K^+^ channel genes was analyzed in RNA samples of 14-day-old seedlings grown on normal 1/2 MS medium.

For real-time PCR assay, total RNA was extracted using the Trizol reagent (Invitrogen) according to the manufacturer’s protocol, and then treated with DNase I (TaKaRa) to eliminate genomic DNA. cDNA was synthesized from treated RNA using SuperScript II reverse transcriptase (Invitrogen). Real-time PCR was performed using SYBR Premix Ex Taq (TaKaRa) on an ABI 7300 real-time PCR system (Applied Biosystems) according to the manufacturer’s instructions. The PCR thermal cycles were as follows: 95°C for 30 s, followed by 40 cycles at 95°C for 5 s and 60°C for 31 s. Relative quantitative results were calculated by normalization to *ACTIN2*. Three replications were performed for each sample. All experiments were repeated three times.

For RT-PCR assay, total RNA was isolated from the *atunc-93* mutants, complementation lines and wild type plants, and then, the expression of *AtUNC-93* was determined by RT-PCR. The Arabidopsis *ACTIN2* gene was used as an internal control.

The primers used in this experiment were listed in Supplementary Table S1.

### Low-K^+^ Stress Treatment and Ion Content Measurement

For low-K^+^ (LK) stress treatment, the K^+^ concentration in the LK medium was 100 μM throughout this study. The LK medium was made by modification of MS medium as described previously (Xu et al., 2006). For the post-germination growth assay on LK medium, 4-day-old seedlings were transferred from MS medium to LK or MS medium, and the low-K^+^ phenotype was observed at 10 days after transfer. For seed germination on LK medium, Arabidopsis seeds were germinated directly on MS, LK, or LK/LNH (low-K^+^ and low-NH_4_^+^) medium for 4 days. The LK/LNH medium was modified from LK medium. Briefly, 28.6 mM NH_4_NO_3_ was removed, and others were unchanged. The actual and final K^+^ concentration in LK/LNH medium was 100 μM, and NH_4_^+^ concentration was 1.25 mM.

For K^+^ and Na^+^ content measurements, 4-day-old Arabidopsis seedlings were transferred from MS medium to LK or MS medium and treated for 10 days. The root and shoot tissues were harvested separately and dried at 80°C for 48 h. The dry weight was measured as an indicator of biomass. Samples were powdered and digested with perchloric acid and nitric acid (1:4) solution. After complete digestion, the acid solution was adjusted to 10 mL with double-distilled water. K^+^ and Na^+^ concentrations of the samples were measured by atomic absorption spectrophotometry. Sixty plants of each line were used for each experiment. All experiments were replicated at least three times.

## Results

### Protein Structure of AtUNC-93

Arabidopsis gene *At3g09470* encodes a protein containing a UNC-93 domain and was named *AtUNC-93*, based on orthology to UNC-93 in *Caenorhabditis elegans* (de la Cruz et al., 2003). No other homologs were found in Arabidopsis. AtUNC-93 appears to be an integral membrane protein possessing ten transmembrane-spanning domains of the deduced AtUNC-93 protein^1^. A Protein BLAST search against the National Center for Biotechnology Information protein database revealed that AtUNC-93 shared high similarity with other UNC-93 orthologs from various species (Supplementary Figure S1). Phylogenetic analysis of the UNC-93 sequences of 18 species indicated that these UNC-93 orthologs were classified from plants and animals separately, and the plants were clustered into two clades, dicotyledons and monocotyledons (Supplementary Figure S2). The strict conservation of these UNC-93 orthologs indicates that they likely play evolutionary conserved roles and have extremely important physiological functions in the different species.

### Expression Pattern and Subcellular Localization of AtUNC-93

**FIGURE 1:**
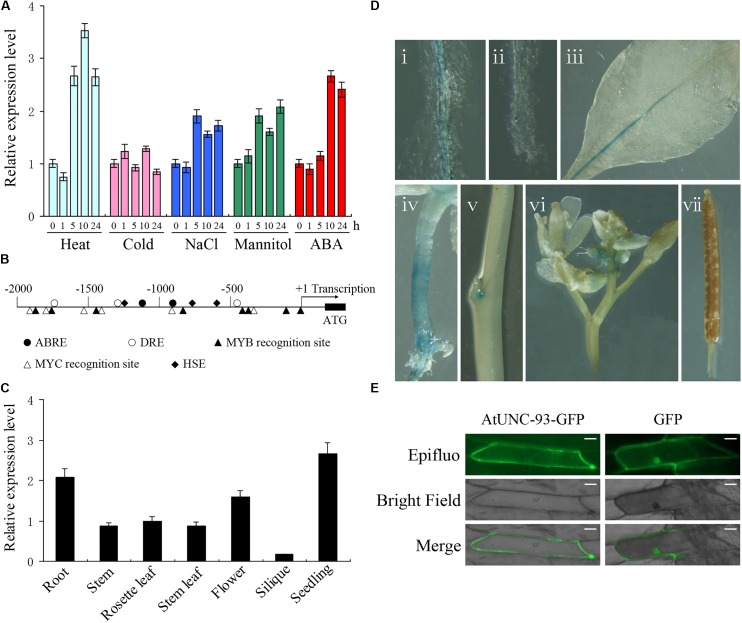
Expression pattern and subcellular localization of AtUNC-93. **(A)** Expression levels of *AtUNC-93* under abiotic stress and ABA treatments. **(B)** Distribution of major stress-related *cis*-elements in the promoter region of *AtUNC-93*. **(C)** Expression levels of *AtUNC-93* expression in different tissues as indicated. **(D)** Expression pattern of *AtUNC-93* determined in *ProAtUNC-93:GUS* transgenic Arabidopsis plants. The GUS staining of primary root (i), primary root tip (ii), leaf (iii), hypocotyl (iv), stem (v), flower (vi), and silique (vii). **(E)** Subcellular localization analysis of AtUNC-93-GFP in onion epidermic cells. The expression of GFP alone was used as controls. Bars = 50 μm. Data in **(A,C)** represent the means ± standard error (SE) (*n* = 3), 3 independent experiments.

Two-week-old seedlings of wild type Arabidopsis were treated with various abiotic stresses and ABA to investigate the expression of *AtUNC-93*. Real-time PCR analysis revealed that *AtUNC-93* expression was significantly induced by heat stress and ABA treatment. By contrast, *AtUNC-93* was only mildly responsive to high salinity and osmotic stress, increasing by ∼2-fold relative to unstressed plants, and was almost not affected by cold stress (**Figure 1A**). The up-regulation of *AtUNC-93* expression by abiotic stresses prompted us to examine its promoter sequence (2,000 bp upstream from the transcription start site) by searching the promoter sequence against the new PLACE database (Higo et al., 1999). The results revealed that the promoter contains many putative stress response-related *cis*-elements (**Figure 1B**), which may be important reason of *AtUNC-93* responding to abiotic stresses.

We also investigated *AtUNC-93* spatial expression patterns in Arabidopsis by real-time PCR. The transcript of *AtUNC-93* was widely detected in all organs tested. *AtUNC-93* expression in the roots and seedlings was very high, but was extremely low in siliques (**Figure 1C**). To gain more insight into the expression patterns of *AtUNC-93*, transgenic Arabidopsis plants expressing β-glucuronidase gene (*GUS*) under control of *AtUNC-93* promoter were generated. GUS activity was strongly detected in the roots, hypocotyls and leaf veins. However, almost no GUS signals were detected in siliques, petals and mesophyll cells (**Figure 1D**). In root tissues, strong GUS staining could be observed in mature root, while the expression was absent in the primary root tip. In shoot tissues, GUS activity was not obviously found in stem, but the expression level of *AtUNC-93* in stem by real-time PCR testing is similar to that in leaf, probably because *AtUNC-93* mainly expressed in vascular bundles but not in stem epidermal cells. The results demonstrate that *AtUNC-93* is preferentially expressed in the vascular tissues of roots, stems and leaves.

The subcellular localization of AtUNC-93 was tested using an AtUNC-93-GFP fusion protein. Transient expression of this fusion protein was examined under confocal microscopy in onion (*Allium cepa*) epidermic cells. AtUNC-93–GFP fluorescence was observed only in the plasma membrane (PM), whereby GFP fluorescence was observed ubiquitously (**Figure 1E**). Thus, we concluded that AtUNC-93 localized to the PM.

### *AtUNC-93* Enhanced Abiotic Stress Tolerance

**FIGURE 2:**
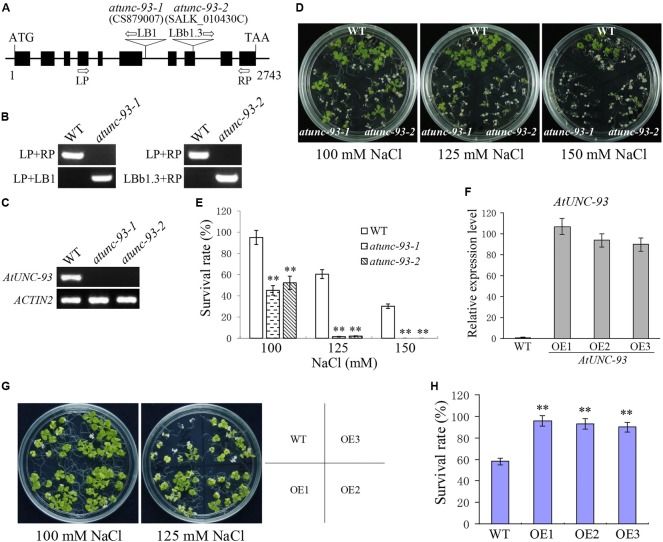
Phenotype tests of wild type, *atunc-93* and *AtUNC-93*-overexpressing seedlings under salt stress. **(A)** Schematic structure of *AtUNC-93* and the positions of the T-DNA insertion of *atunc-93-1* and *atunc-93-2*. The boxes indicate exons, and the lines represent introns and untranslated regions. **(B)** Genomic identification of *atunc-93-1* and *atunc-93-2*. LP, left primer; RP, right primer; LB1 and LBb1.3, the T-DNA left border primer. WT is the wild type. **(C)** Reverse transcription (RT)-PCR verification of *AtUNC-93* expression in *atunc-93* mutants. **(D,E)** Phenotype **(D)** and the survival rates **(E)** of *atunc-93* mutant seedlings and the wild type grown on 1/2 MS medium containing 100, 125, and 150 mM NaCl for 14 days, respectively. **(F)** Real-time PCR verification of *AtUNC-93* in *35S:AtUNC-93* transgenic lines (OE1, OE2, and OE3). **(G,H)** Phenotype **(G)** and the survival rates **(H)** of *AtUNC-93*-overexpressing lines and the wild type grown on 1/2 MS medium containing 100 and 125 mM NaCl for 14 days, respectively. Data in **(E,F,H)** represent the means ± SE (*n* = 3). The student’s *t*-test (^∗∗^*P* < 0.01) was used to analyze statistical significance compared with the wild type.

The *AtUNC-93* T-DNA insertion mutants, *atunc-93-1* and *atunc-93-2*, were used to reveal the function of AtUNC-93. The T-DNA insertion sites of *atunc-93-1* and *atunc-93-2* are located in the sixth intron and the eighth exon of *AtUNC-93* gene, respectively (**Figure 2A**), which were confirmed by PCR analysis and sequencing the amplified fragments (**Figure 2B**). Reverse transcription (RT)-PCR analysis showed that *AtUNC-93* expression was abolished in these two homozygous *atunc-93* mutants (**Figure 2C**). We observed that the *atunc-93-1* and *atunc-93-2* plants grown under standard growth conditions showed retarded growth and no other obvious phenotypic difference compared with the wild type. But under salt stress conditions, the cotyledons of most *atunc-93-1* and *atunc-93-2* seedlings were bleached with 100 mM NaCl treatment, whereas only few wild type seedlings were impaired (**Figures 2D,E**). Even under the treatment of 150 mM NaCl, all *atunc-93-1* and *atunc-93-2* seedlings were completely dead, but about 30% of wild type seedlings still survived (**Figures 2D,E**). These results showed that disruption of *AtUNC-93* resulted in decreased salt stress tolerance at seedling stage. To further reveal the role of *AtUNC-93* in response to salt stress, *AtUNC-93*-overexpressing lines were generated, and the expression levels of *AtUNC-93* in these lines were much higher than in the wild type (**Figure 2F**). After 125 mM NaCl treatment for 14 days, the survival rate of wild type plants fell to about 58%, whereas nearly 100% of *AtUNC-93*-overexpressing seedlings were alive (**Figures 2G,H**). These results suggested that overexpression of *AtUNC-93* significantly enhanced salt tolerance of transgenic lines at seedling stage and three independent overexpression lines showed similar results. Data of the independent overexpression line OE1 and OE2 are shown in afterward analysis. To directly discern salt tolerance differences of *atunc-93* mutants and overexpression lines, all materials were tested in the same plate. This finding was consistent with the fact that *atunc-93* mutants were more susceptible to salt stress, whereas the overexpression lines improved salt tolerance (Supplementary Figures S3A,C). The complementation lines showed the wild type salt tolerance inclination (Supplementary Figures S4B,D), indicating that the hypersensitivity to salt stress of *atunc-93* mutants is due to the loss of function of *AtUNC-93*.

**FIGURE 3:**
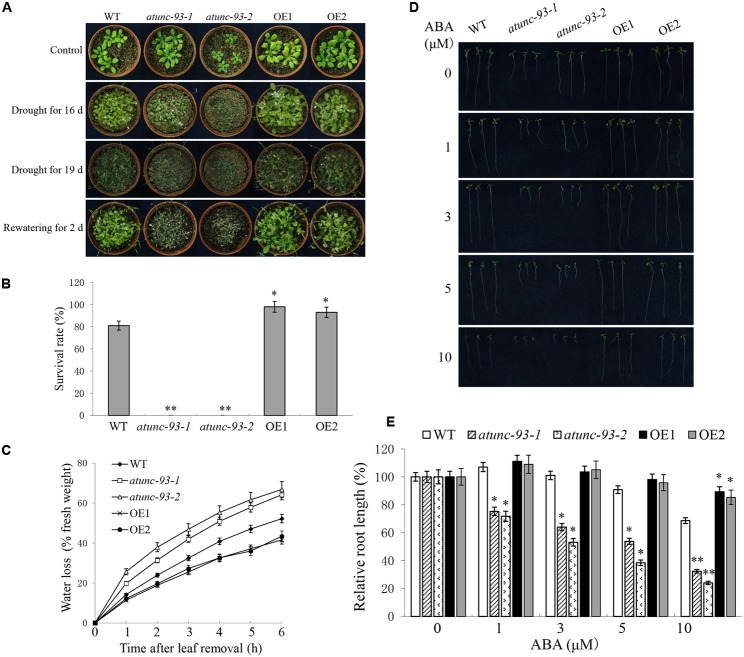
Wild type, *atunc-93* and *AtUNC-93*-overexpressing seedlings under drought stress and ABA treatment. **(A)** Phenotype comparison of wild type, *atunc-93* and *AtUNC-93*-overexpressing seedlings under drought stress. Two-week-old seedlings were grown for 16 or 19 days without irrigation, and then were rewatered for 2 days. Irrigated seedlings were used as controls. The experiments were repeated three times, with similar results. **(B)** The survival rates of various plant materials were determined as the number of visible green plants after rehydration. **(C)** Quantitative determination of water loss of detached leaves. **(D)** Seedlings grown in medium lacking or supplemented with ABA for 7 days. **(E)** Relative root length of seedlings based on panel **(D)**. Forty plants of each line were used for each experiment. Data in **(B,C,E)** are shown as means ± SE (*n* = 3), 3 independent experiments. Student’s *t*-test (^∗^*P* < 0.05 and ^∗∗^*P* < 0.01) was used to analyze statistical significance compared with the wild type.

Salt-tolerant role of *AtUNC-93* led us to check the tolerance to other abiotic stresses. For drought stress, two-week-old seedlings grown on soil pots were subjected to drought stress by stopping watering. Sixteen days later *atunc-93-1* and *atunc-93-2* plants showed severe wilting and chlorosis, and wild type plants showed weak chlorosis, whereas no obvious damage was observed in the *AtUNC-93*-overexpressing plants (**Figure 3A**). More severe wilting symptoms were observed in nearly all plants as the drought stress was prolonged for 3 days again (**Figure 3A**). After rewatering for 2 days, about 80% wild type plants were able to survive and continued to grow. By contrast, all of *atunc-93-1* and *atunc-93-2* plants did not recover from the stress and died. Almost all of the *AtUNC-93*-overexpressing plants were survived and showed more vigorous growth than the wild type (**Figures 3A,B**). Moreover, the water loss from detached leaves was increased in the *atunc-93* mutant plants compared with the wild type, while the *AtUNC-93*-overexpressing plants reduced water loss (**Figure 3C**). These results indicated that *AtUNC-93* may function as a positive regulator in response to drought stress. Osmotic stress treatments were also performed at seedling stage. The *atunc-93* mutant plants became chlorotic and died under the treatment with mannitol (Supplementary Figures S3B,D). In contrast, the wild type and *AtUNC-93*-overexpressing plants still remained more green leaves and higher survival rates, and *AtUNC-93*-overexpressing plants grew better than wild type plants after mannitol treatment (Supplementary Figures S3B,D). The complementation lines of *atunc-93-1* and *atunc-93-2* mutants rescued sensitive phenotype of these mutant seedlings to osmotic stress (Supplementary Figures S4C,E).

Thermotolerance of *atunc-93* mutants and *AtUNC-93*-overexpressing plants was further investigated. First, we tested them for basal thermotolerance at 45°C. The *atunc-93* mutant plants showed more severe inhibition in shoot growth when directly exposed to 45°C for 2 h (Supplementary Figure S5A). Next, we measured the acquired thermotolerance using two strategies. One was that seedlings were acclimated for 2 h at 38°C and immediately subjected to 45°C. After 3 h treatment at 45°C, *atunc-93-1* and *atunc-93-2* plants were severely injured, but wild type plants showed no observable symptoms. When plants were continuously subjected to 45°C for 4 h, *atunc-93-1* and *atunc-93-2* plants nearly completely died, but half of wild type plants survived (Supplementary Figure S5A). The other strategy was that seedlings were recovered for 2 h at the normal temperature (22°C) between acclimation (38°C) and lethal treatment (45°C). When lethal heat treatment was imposed for 4 h in this strategy, *atunc-93-1* and *atunc-93-2* plants suffered severe damage, but wild type plants survived, which is similar to the result after 3 h of lethal heat treatment without recovery period (Supplementary Figure S5A). The two strategies in heat treatment were manifested that recovery period between acclimation and lethal treatment can retard heat-damage. In all thermotolerance assays, *atunc-93* mutants decreased the basal and acquired thermotolerance, but no differences were observed between *AtUNC-93*-overexpressing plants and the wile type. The *atunc-93* mutants, *AtUNC-93*-overexpressing lines and the wild type were also tested for chilling-tolerance at seedling stage, but no significant difference was observed (Supplementary Figure S5B).

### The *atunc-93* Mutants Are Hypersensitive to ABA

Abscisic acid is an important hormone for abiotic stress in plants (Finkelstein et al., 2002). Most experimental evidences were provided for the involvement of bZIP transcription factors and ion channels in regulation of abiotic stress tolerance by ABA signaling (Diaz et al., 2016; Zong et al., 2016). In this experiment, *atunc-93-1* and *atunc-93-2* plants showed ABA hypersensitive phenotypes in ABA-induced post-germination growth inhibition. The root growth of *atunc-93-1* and *atunc-93-2* was more severely inhibited as the ABA concentration increased. The relative root length (the root length in the ABA treatment to that in the ABA-free medium) of *atunc-93-1* and *atunc-93-2* was significantly decreased to 32 and 24% under the treatment with 10 μM ABA, respectively (**Figures 3D,E**). The *AtUNC-93*-overexpressing plants exhibited high relative root length (89 and 85%) compared with the wild type (69%) after treatment with 10 μM ABA (**Figures 3D,E**). These results suggested that disruption of *AtUNC-93* in plants generated hypersensitivity to ABA, while overexpression of *AtUNC-93* in plants resulted in decreased sensitivity to ABA.

### The Expression of Stress-Related and ABA-Responsive Genes Is Affected by the Transcript Level of *AtUNC-93*

**FIGURE 4:**
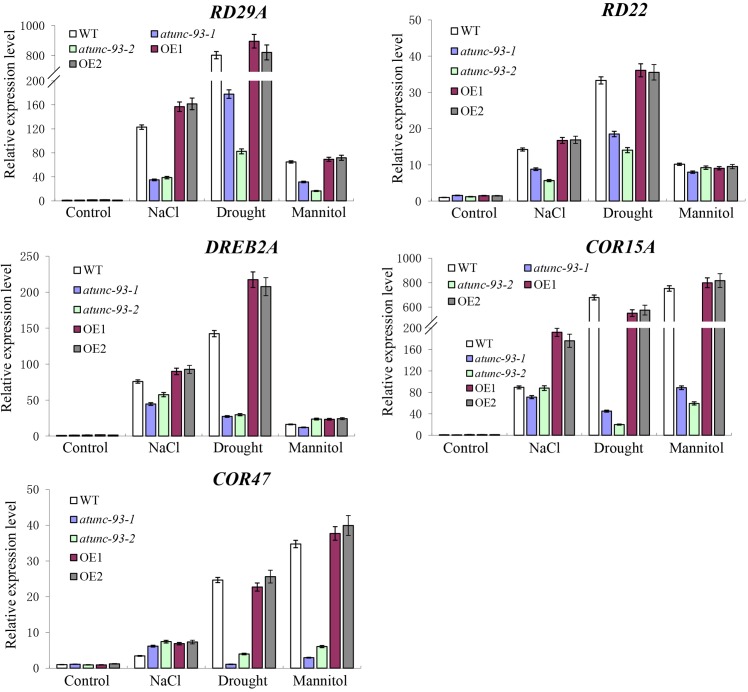
Expression of the stress-related genes in *atunc-93* mutants and *AtUNC-93*-overexpressing lines under stress conditions. The expression levels of *RD29A, RD22, DREB2A, COR15A*, and *COR47* were determined by using real-time PCR under NaCl, drought and mannitol treatments. Seedlings before stress treatments were used as controls. Each data bar represents the mean ± SE (*n* = 3).

Many studies have reports that plant tolerance to abiotic stress is involved in the transcription regulation of a number of stress-related genes (Yamaguchi-Shinozaki and Shinozaki, 2006; Shi et al., 2017). Then we analyzed expression levels of some stress-related genes, such as *RD29A, RD22, DREB2A, COR15A*, and *COR47*, under stress conditions. All these genes were induced by various abiotic stresses in wild type plants (**Figure 4**). Moreover, the expression levels of these genes were significantly lower in *atunc-93* mutants and higher in *AtUNC-93*-overexpressing plants than in the wild type under stress conditions (**Figure 4**). These results suggested that expression level changes of the stress-related genes in *atunc-93* mutants and *AtUNC-93*-overexpressing plants under stress conditions may contribute to the significant difference of stress tolerance.

**FIGURE 5:**
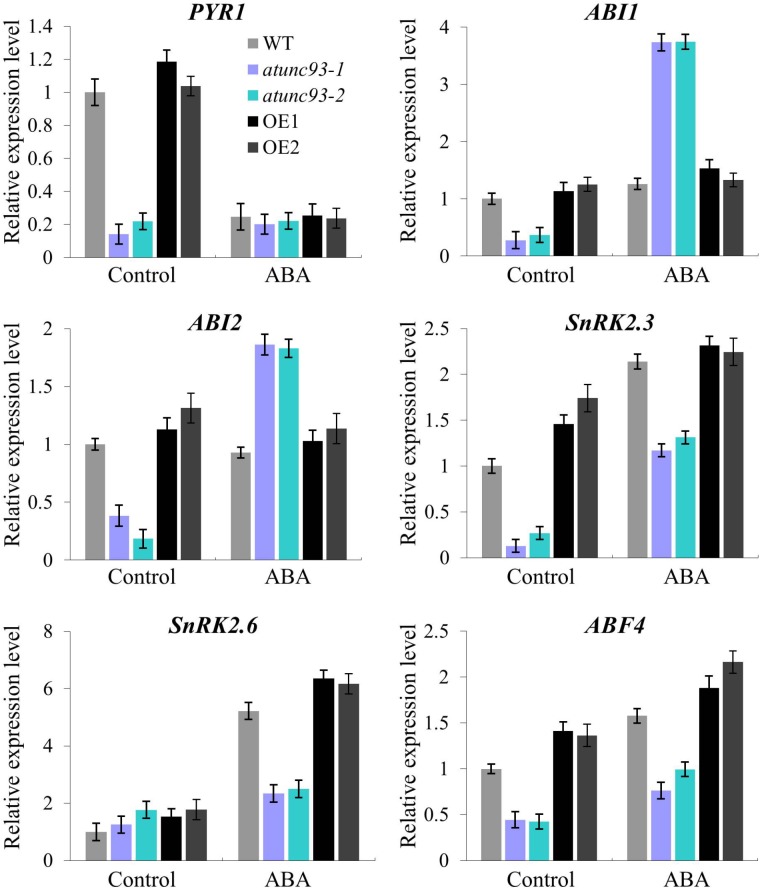
Expression of the core component genes in ABA signaling pathway in *atunc-93* mutants and *AtUNC-93*-overexpressing lines. Relative expression of *PYR1, ABI1, ABI2, SnRK2.3, SnRK2.6*, and *ABF4* was analyzed by using real-time PCR under the treatments with or without ABA. Data represent the means ± SE (*n* = 3).

Next, the ABA hypersensitivity of *atunc-93* mutants prompted us to check the expression of ABA-responsive genes in *atunc-93* mutants and *AtUNC-93*-overexpressing plants. Under normal growth conditions, the expression levels of the core component genes of ABA signaling pathway, including *PYR1, ABI1, ABI2, SnRK2.3*, and *ABF4*, were apparently down-regulated in the *atunc-93* mutants compared with the wild type (**Figure 5**). However, the negative regulator genes, *ABI1* and *ABI2*, were significantly up-regulated by exogenous ABA application in the *atunc-93* mutants compared with the wild type (**Figure 5**). Meanwhile, the expressions of positive regulators (SnRK2.3, SnRK2.6, and ABF4) were also up-regulated by exogenous ABA, but the up-regulation level was strongly suppressed in the *atunc-93* mutants (**Figure 5**). This result demonstrated that the expressions of *ABI1, ABI2, SnRK2.3, SnRK2.6*, and *ABF4* are modulated by *AtUNC-93*. It is noteworthy that, the expression of *PYR1* was not changed by ABA in the *atunc-93* mutants, but was significantly down-regulated in the wild type and decreased to the transcript level in the *atunc-93* mutants after ABA treatment (**Figure 5**), suggesting that the transcript of *PYR1* is also regulated by *AtUNC-93*. The expression of these genes was also tested in the *AtUNC-93*-overexpressing lines, but no significant difference was observed between the *AtUNC-93*-overexpressing lines and the wild type under the treatment with or without ABA (**Figure 5**). In addition, the expression levels of *DREB1A* and *DREB2A* were significantly lower in the *atunc-93* mutants and higher in the *AtUNC-93*-overexpressing lines than those in the wild type with ABA treatment (Supplementary Figure S6). *MYB2* expression was markedly repressed in the *atunc-93* mutants without ABA treatment (Supplementary Figure S6). However, the expression levels of other ABA-responsive genes in this study were not changed much in the *atunc-93* mutants and the *AtUNC-93*-overexpressing lines compared with the wild type, though some differences were still detected.

### AtUNC-93 Positively Controlled Plant Growth

**FIGURE 6:**
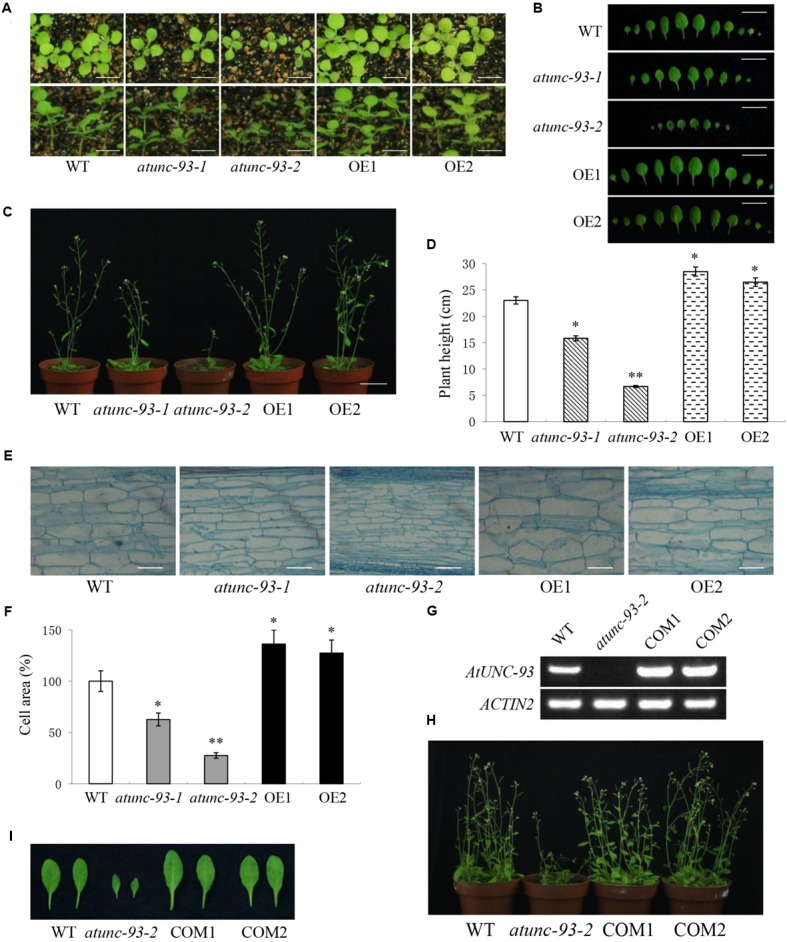
Plant growth phenotypes of the wild type, *atunc-93* mutants and *AtUNC-93*-overexpressing plants. **(A)** 14-day-old seedlings grown in soil pots. Bars = 5 mm. **(B)** Rosette leaves of 21-day-old seedlings. Bars = 10 mm. **(C,D)** Phenotype **(C)** and plant height **(D)** of 6-week-old plants. Bars = 30 mm. **(E,F)** Stem cells comparison **(E)** and cell areas **(F)** of 6-week-old plants. Bars = 100 μm. Relative cell areas were calculated from the microscope image data using ImageJ. **(G)** RT-PCR verification of *AtUNC-93* expression levels in the wild type, *atunc-93-2* and complementation lines (COM1 and COM2). **(H,I)** Phenotype comparison **(H)** and rosette leaves **(I)** of the wild type, *atunc-93-2* and complementation plants grown in soil pots for 6 weeks. Data in **(D,F)** are shown as means ± SD (*n* = 30), thirty plants or cells were tested. Student’s *t*-test (^∗^*P* < 0.05 and ^∗∗^*P* < 0.01) was used to analyze statistical significance compared with the wild type.

When grown in soil, *atunc-93-1* and *atunc-93-2* plants showed dwarf phenotypes compared with wild type plants, especially for *atunc-93-2*, while *AtUNC-93*-overexpressing plants exhibited larger bodies than wild type plants (**Figures 6A,C**). Along with plants growth and development, the difference significance of growth phenotypes increased gradually. The differences of leaves size (**Figure 6B**) and plants height (**Figure 6D**) among all lines were in accord with phenotypes. Previous studies have shown that cell expansion plays an essential role in plant growth (Zonia and Munnik, 2007; Osakabe et al., 2013). Then we observed that the stem cells from 6-week-old plants were smaller in *atunc-93-1* and *atunc-93-2*, larger in *AtUNC-93*-overexpressing plants, than in the wild type (**Figures 6E,F**). These observations implied that the phenotype differences of *atunc-93* mutants and the *AtUNC-93*-overexpressing plants were resulted from alterations of cell expansion.

We also observed that the length of roots and hypocotyls of *atunc-93-1* and *atunc-93-2* was significantly shorter than that of the wild type (Supplementary Figures S7A–C). Interestingly, *atunc-93-1* had longer hypocotyls but shorter roots than those of *atunc-93-2*. Meanwhile *AtUNC-93*-overexpressing lines showed a little longer hypocotyls and roots compared with the wild type (Supplementary Figures S7A–C). Especially *AtUNC-93*-overexpressing plants showed more root biomass than that in wild type plants when grown for 2 weeks (Supplementary Figure S7E), which suggested that *AtUNC-93*-overexpressing plants exhibited rapid growth phenotype due to its extensive root system.

To further confirm the role of *AtUNC-93* involved in plant growth, we observed the growth phenotype and leaf size in the complementation lines of *atunc-93-2*. It had been shown that the complementation lines rescued the inhibited growth phenotype of *atunc-93-2* (**Figures 6H,I**). Thus, *atunc-93* mutants showed dwarf phenotypes were caused by disruption of the *AtUNC-93* gene.

### AtUNC-93 Regulates K^+^ Accumulation in Arabidopsis Shoots

**FIGURE 7:**
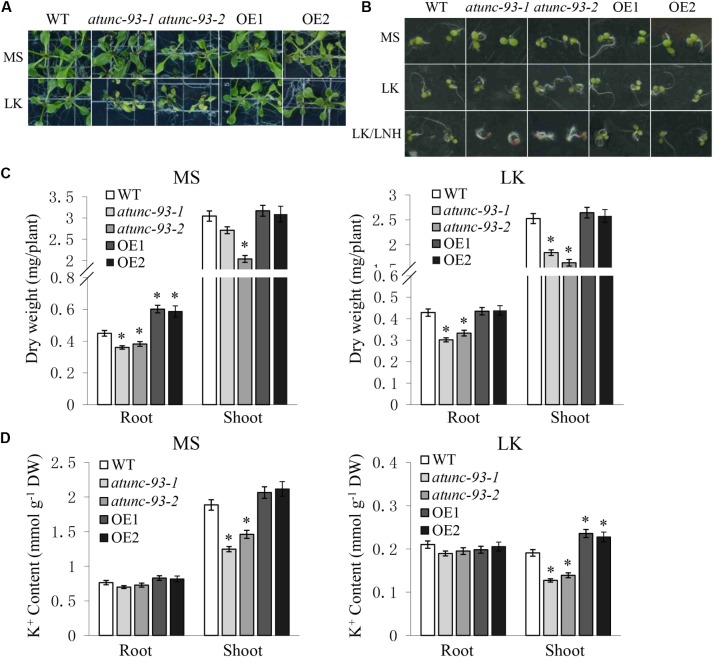
Phenotype tests and K^+^ content measurements of the wild type, *atunc-93* mutants and *AtUNC-93*-overexpressing lines under low-K^+^ conditions. **(A)** Phenotype of various materials after being transferred to MS or LK medium for 10 days. MS indicates Murashige and Skoog medium (20 mM K^+^, 20 mM NH_4_^+^). LK indicates modified MS medium containing low-K^+^ concentration (100 μM K^+^, 30 mM NH_4_^+^). **(B)** Phenotype comparison of various materials after the seeds germinated directly on MS, LK or LK/LNH medium for 4 days. MS and LK indicate as shown in **(A)**. LK/LNH indicates modified MS medium containing low-K^+^ and low-NH_4_^+^ concentration (100 μM K^+^, 1.25 mM NH_4_^+^). **(C,D)** Dry weight **(C)** and K^+^ content **(D)** of various plant materials as indicated under MS and LK conditions. Data are shown as means ± SE (*n* = 3). Student’s *t*-test (^∗^*P* < 0.05) was used to analyze statistical significance compared with the wild type.

UNC-93 was defined as a regulatory protein of the SUP-9 two-pore K^+^ channel in *Caenorhabditis elegans* and conserved among *Caenorhabditis elegans*, humans and *Arabidopsis thaliana* (Levin and Horvitz, 1992; de la Cruz et al., 2003), suggesting that UNC-93-like regulatory proteins represent a conserved mechanism for the regulation of K^+^ transport. This finding led us to hypothesize that AtUNC-93 may also regulate K^+^ uptake and/or translocation in Arabidopsis. To verify this hypothesis, phenotype observation and K^+^ content measurements of *atunc-93* mutants, *AtUNC-93*-overexpressing lines and the wild type under normal (MS medium, 20 mM K^+^) and low-K^+^ (LK medium, 100 μM K^+^) growth conditions were performed. The leaves of *atunc-93-1* and *atunc-93-2* became chlorotic, and their growth was suppressed significantly under low-K^+^ conditions (**Figure 7A**). The phenotypes of leaf chlorosis and inhibition of growth are taken as typical symptoms under K^+^-deficient conditions (Xu et al., 2006). The low-K^+^ sensitive phenotypes of *atunc-93* mutants were also found below 500 μM external K^+^ concentrations. But along with the external K^+^ concentrations rising, the sensitive phenotypes of *atunc-93* mutants disappeared gradually (Supplementary Figure S8A). In addition, we also tested the seed germination under low-K^+^ conditions. Because the presence of millimolar NH_4_^+^ is necessary for low-K^+^ sensitive phenotype of plants at germination stage (Hirsch et al., 1998; Spalding et al., 1999), we planted the seeds directly in medium supplemented with various concentrations of K^+^ and NH_4_^+^. After incubated for 4 days, the *atunc-93* mutants exhibited no significant difference from the wild type under low-K^+^ (100 μM K^+^, 30 mM NH_4_^+^) conditions (**Figure 7B**). Under low-K^+^ and low-NH_4_^+^ (100 μM K^+^, 1.25 mM NH_4_^+^) conditions, *atunc-93-1* and *atunc-93-2* showed brown cotyledons, inhibited primary root growth and stimulated root hair elongation, which were typical K^+^-deficient symptoms (Jung et al., 2009; Tsay et al., 2011). But these were not detected in *AtUNC-93*-overexpressing lines and wild type plants. These results indicated that *atunc-93* mutants displayed hypersensitivity to low-K^+^ stress in both seed germination and post-germination growth.

To quantify K^+^-dependent plant growth, the biomass measurements were determined. The *atunc-93-1* and *atunc-93-2* seedlings showed less dry weight of roots and shoots under low-K^+^ conditions than those under normal growth conditions, which were in agreement with their grow phenotype (**Figure 7C**). In addition, the dry weight of roots of *AtUNC-93*-overexpressing lines was remarkably higher than that of wild type plants under normal growth conditions, but was not significantly different from that of wild type plants under low-K^+^ conditions (**Figure 7C**), which further demonstrated that overexpression of *AtUNC-93* gene may promote root growth in Arabidopsis under normal growth conditions.

The shoot K^+^ content of *atunc-93-1* and *atunc-93-2* was much lower than that of the *AtUNC-93*-overexpressing lines and wild type plants after cultured on both MS and LK medium (**Figure 7D**). The *AtUNC-93*-overexpressing lines showed significantly higher K^+^ content in shoots compared with the wild type under low-K^+^ conditions, but no significant difference was observed under the normal growth conditions (**Figure 7D**). Additionally, there was no significant difference in root K^+^ content among the all tested materials under either normal or low-K^+^ conditions (**Figure 7D**). In the meantime, we also detected Na^+^ contents. The results of Na^+^ content measurement indicated that all tested materials had similar Na^+^ contents (Supplementary Figure S9A). The K^+^/Na^+^ ratios were significantly lower for the shoots of *atunc-93* mutants compared with the *AtUNC-93*-overexpressing lines and wild type plants under both normal and low-K^+^ conditions. The *AtUNC-93*-overexpressing lines exhibited higher K^+^/Na^+^ ratios for the shoots compared with the wild type under low-K^+^ conditions (Supplementary Figure S9B). All of these results suggested that *AtUNC-93* gene in Arabidopsis may play a crucial role in translocating K^+^ from roots to shoots. This may explain the K^+^-deficient symptoms of *atunc-93* mutants under low-K^+^ stress. To investigate whether AtUNC-93 participates in the regulation of K^+^ transport in Arabidopsis by changing the transcript levels of K^+^ channels, the expression of 15 K^+^ channel genes were detected. Whereas no significant difference was tested among all the materials (Supplementary Figure S8B), indicating that the regulation of K^+^ transport by AtUNC-93 may not occur at the transcriptional alteration of K^+^ channels. AtUNC-93 plays an important role in the regulation of K^+^ transport in Arabidopsis. K^+^ homeostasis is a key factor for plant in response to abiotic stresses and growth. Then, these results together suggest that AtUNC-93 regulates abiotic stress tolerance and growth by maintaining K^+^ homeostasis in Arabidopsis.

## Discussion

K^+^ is one of the principal nutrient elements for plant growth and development. Many reports have shown that the mutants of K^+^ channels or regulators exhibit leaf chlorosis, inhibition of growth and lower K^+^ contents under low-K^+^ conditions, which are K^+^-deficient symptoms (Xu et al., 2006; Li et al., 2017; Wang and Wu, 2017). In this study, the *atunc-93* mutants showed significant LK-sensitive phenotype at both germination and post-germination stages and lower K^+^ contents in shoots compared with the wild type, while no obvious difference of the root K^+^ content was observed (**Figure 7**). It is suggested that AtUNC-93 participates in the response to K^+^ deficiency and is involved in the root-to-shoot K^+^ translocation. The outward-rectifying K^+^ channel SKOR is localized in root stelar tissues and contributes to K^+^ translocation from roots to shoots (Gaymard et al., 1998). The expression of *AtUNC-93* was detected mainly in the vascular tissues (**Figure 1D**), suggesting that AtUNC-93 may have the similar role as SKOR in K^+^ translocation toward the shoots. This regulation of K^+^ translocation from roots to shoots was recently reported in Arabidopsis by transporter NRT1.5 and in rice by outward Shaker K^+^ channel OsK5.2 (Li et al., 2017; Nguyen et al., 2017). However, the *AtUNC-93*-overexpressing plants did not show significant phenotype differences compared with wild type plants (**Figures 7A,B**). Only the shoot K^+^ contents of the *AtUNC-93*-overexpressing plants were much higher than those of wild type plants under low-K^+^ conditions (**Figure 7D**). The possible reason is that the expression levels of *AtUNC-93* in the wild type are sufficient to maintain K^+^ homeostasis in plant cells under normal growth conditions so that no significant difference of the shoot K^+^ contents was detected between the *AtUNC-93*-overexpressing plants and the wild type. But under low-K^+^ conditions, more amounts of AtUNC-93 are needed to promote the K^+^ translocation from roots to shoots. The similar result has also been reported for CBL10, which is involved in K^+^ homeostasis by negatively regulating AKT1 activity in Arabidopsis. The *CBL10*-overexpressing lines showed a phenotype as sensitive as that of the *akt1* mutant under low-K^+^ conditions, while the *cbl10* mutant did not show significant phenotype differences compared with wild type plants (Ren et al., 2013). AtUNC-93 is identified to be a member of major facilitator superfamily (MFS), which is a large and diverse group of secondary transporters. The main function of MFS proteins is to facilitate the transport across cytoplasmic or internal membranes of a variety of substrates including ions, nucleosides, amino acids, and so on (Marchler-Bauer, 2015). The MFS transporter ZIFL2 promotes cellular K^+^ efflux in the root and regulates K^+^ translocation from roots to shoots in Arabidopsis under conditions of high K^+^ external supply (Remy et al., 2015).

The maintenance of cellular K^+^ homeostasis will contribute to improving plant tolerance to abiotic stresses. Recent studies reported that two K^+^ channels OsAKT1 and OsTPKb play pivotal roles in osmotic and drought stress tolerance by maintaining cellular K^+^ homeostasis (Ahmad et al., 2016a,b). In this study, *AtUNC-93* was induced by salt, osmotic and heat stress (**Figure 1A**). Overexpression of *AtUNC-93* in Arabidopsis significantly enhanced salt, drought and heat tolerance during seedling development, while disruption of *AtUNC-93* resulted in lower stress tolerance (**Figures 2, 3** and Supplementary Figures S3–5). This finding suggests that AtUNC-93 may play a critical role in stress tolerance by maintaining K^+^ homeostasis in plants. K^+^, as osmotically active substance, can regulate cell turgor pressure, which facilitates cell expansion and leads to plant growth (Wang and Wu, 2013). KUP6 acts as a key factor in K^+^ homeostasis and negatively regulates turgor-dependent growth (Osakabe et al., 2013). Our study reveals that the *atunc-93* mutant plants exhibited dwarf phenotypes, while *AtUNC-93*-overexpressing transgenic plants had large plant bodies (**Figure 6** and Supplementary Figure S7). The changes of vegetative growth resulted from the alteration of cell expansion, suggesting that AtUNC-93 positively modulate turgor-dependent growth through cellular K^+^ homeostasis.

Abscisic acid is produced rapidly in response to abiotic stress and plays a crucial role in the modulation of stress responses. AtHSPR, a heat shock protein-related, is involved in salt and drought stress tolerance in Arabidopsis by maintaining K^+^ /Na^+^ homeostasis in ABA-dependent pathways (Yang et al., 2015). In this study, the expression of *AtUNC-93* was up-regulated by ABA treatment (**Figure 1A**). On the other hand, *AtUNC-93* also regulated the expression of a set of ABA-responsive genes (**Figure 5** and Supplementary Figure S6). Under no exogenous ABA conditions, the expression levels of *PYR1, ABI1, ABI2, SnRK2.3*, and *ABF4* were apparently down-regulated in the *atunc-93* mutants compared with the wild type. The transcript abundance of ABA receptor gene *PYR1* in the *atunc-93* mutants under no exogenous ABA conditions was similar to that in the wild type under ABA treatment (**Figure 5**), suggesting that *atunc-93* mutants under normal conditions has same physiological responses of the wild type under ABA treatment. But with the exogenous ABA treatment, the ABA signaling negative regulator-encoding genes, *ABI1* and *ABI2*, were strongly induced, and positive regulator-encoding genes, *SnRK2.3, SnRK2.6*, and *ABF4*, were obviously repressed in the *atunc-93* mutants compared with the wild type (**Figure 5**). These results demonstrate that AtUNC-93 functions as a negative regulator in the ABA signaling pathway of the PYR/PYL/RCARs-PP2Cs-SnRK2s regulatory module (Fujii et al., 2009), and regulates *PYR1, ABI1, ABI2, SnRK2.3, SnRK2.6*, and *ABF4* expression. In the phenotype tests, *atunc-93* mutants showed the strongest ABA hypersensitive phenotypes in post-germination growth, while the *AtUNC-93*-overexpressing plants were ABA-insensitive in terms of root growth (**Figure 3D**). It is also indicated that AtUNC-93 is a negative regulator in ABA signaling. The *atunc-93* mutants both increased the sensitivity to ABA and decreased salt, drought, and heat stress tolerance during seedling development. Meanwhile, the *AtUNC-93*-overexpressing plants displayed ABA-insensitive phenotypes as well as enhanced tolerance to salt, drought, and heat stress. Considering ABA can mediate diverse aspects of physiological responses to environmental stresses such as salt, drought, heat, and cold stress (Finkelstein et al., 2002), AtUNC-93 may be a major player in mediating ABA-dependent pathways for conferring stress tolerance.

## Conclusion

In conclusion, AtUNC-93 promotes K^+^ translocation from roots to shoots in Arabidopsis and positively regulates salt, drought and heat stress tolerance and turgor-dependent growth. These stresses tolerance and plant growth are involved in maintaining K^+^ homeostasis through ABA-dependent signal transduction pathways. AtUNC-93 provides a novel important component to examine the ABA-dependent regulatory networks in stress responses and plant growth.

## Author Contributions

XC conceived the study. JX performed all the experiments, analyzed the data and wrote the manuscript. XyZ, XwZ, AL, and YP participated in the experiment. XC, YX, and MY reviewed and edited the manuscript. All authors read and approved the manuscript for final submission.

## Conflict of Interest Statement

The authors declare that the research was conducted in the absence of any commercial or financial relationships that could be construed as a potential conflict of interest.

## Footnotes

1 http://www.cbs.dtu.dk/services/TMHMM-2.0/

## References

[B1] Abdelaziz M. E. Kim D. Ali S. Fedoroff N. V. Al-Babili S. (2017). The endophytic fungus *Piriformospora indica* enhances *Arabidopsis thaliana* growth and modulates Na^+^/K^+^ homeostasis under salt stress conditions. *Plant Sci.* 263 107–115. 10.1016/j.plantsci.2017.07.006 28818365

[B2] Ahmad I. Devonshire J. Mohamed R. Schultze M. Maathuis F. J. (2016a). Overexpression of the potassium channel TPKb in small vacuoles confers osmotic and drought tolerance to rice. *New Phytol.* 209 1040–1048. 10.1111/nph.13708 26474307

[B3] Ahmad I. Mian A. Maathuis F. J. (2016b). Overexpression of the rice AKT1 potassium channel affects potassium nutrition and rice drought tolerance. *J. Exp. Bot.* 67 2689–2698. 10.1093/jxb/erw103 26969743 PMC4861017

[B4] Bassil E. Ohto M. A. Esumi T. Tajima H. Zhu Z. Cagnac O. (2011). The Arabidopsis intracellular Na+/H+ antiporters NHX5 and NHX6 are endosome associated and necessary for plant growth and development. *Plant Cell* 23 224–239. 10.1105/tpc.110.079426 21278129 PMC3051250

[B5] Chen J. Zhang H. Zhang X. Tang M. (2017). Arbuscular mycorrhizal symbiosis alleviates salt stress in black locust through improved photosynthesis, water status, and K^+^/Na^+^ Homeostasis. *Front. Plant Sci.* 8:1739. 10.3389/fpls.2017.01739 29067036 PMC5641402

[B6] Clough S. J. Bent A. F. (1998). Floral dip: a simplified method for *Agrobacterium*-mediated transformation of *Arabidopsis thaliana*. *Plant J.* 16 735–743. 10.1046/j.1365-313x.1998.00343.x 10069079

[B7] de la Cruz I. P. Levin J. Z. Cummins C. Anderson P. Horvitz H. R. (2003). sup-9, sup-10, and unc-93 may encode components of a two-pore K^+^ channel that coordinates muscle contraction in *Caenorhabditis elegans*. *J. Neurosci.* 23 9133–9145. 10.1523/JNEUROSCI.23-27-09133.2003 14534247 PMC6740817

[B8] Diaz M. Sanchez-Barrena M. J. Gonzalez-Rubio J. M. Rodriguez L. Fernandez D. Antoni R. (2016). Calcium-dependent oligomerization of CAR proteins at cell membrane modulates ABA signaling. *Proc. Natl. Acad. Sci. U.S.A.* 113 E396–E405. 10.1073/pnas.1512779113 26719420 PMC4725540

[B9] Finkelstein R. R. Gampala S. S. Rock C. D. (2002). Abscisic acid signaling in seeds and seedlings. *Plant Cell* 14 S15–S45. 10.1105/tpc.010441 12045268 PMC151246

[B10] Fujii H. Chinnusamy V. Rodrigues A. Rubio S. Antoni R. Park S. Y. (2009). In vitro reconstitution of an abscisic acid signalling pathway. *Nature* 462 660–664. 10.1038/nature08599 19924127 PMC2803041

[B11] Gaymard F. Pilot G. Lacombe B. Bouchez D. Bruneau D. Boucherez J. (1998). Identification and disruption of a plant shaker-like outward channel involved in K+ release into the xylem sap. *Cell* 94 647–655. 10.1016/S0092-8674(00)81606-2 9741629

[B12] Higo K. Ugawa Y. Iwamoto M. Korenaga T. (1999). Plant cis-acting regulatory DNA elements (PLACE) database: 1999. *Nucleic Acids Res.* 27 297–300. 10.1093/nar/27.1.297 9847208 PMC148163

[B13] Hirsch R. E. Lewis B. D. Spalding E. P. Sussman M. R. (1998). A role for the AKT1 potassium channel in plant nutrition. *Science* 280 918–921. 10.1126/science.280.5365.918 9572739

[B14] Jefferson R. A. Kavanagh T. A. Bevan M. W. (1987). GUS fusions: β-glucuronidase as a sensitive and versatile gene fusion marker in higher plants. *EMBO J.* 6 3901–3907. 3327686 10.1002/j.1460-2075.1987.tb02730.x PMC553867

[B15] Jung J. Y. Shin R. Schachtman D. P. (2009). Ethylene mediates response and tolerance to potassium deprivation in *Arabidopsis*. *Plant Cell* 21 607–621. 10.1105/tpc.108.063099 19190240 PMC2660615

[B16] Kim B. G. Waadt R. Cheong Y. H. Pandey G. K. Dominguez-Solis J. R. Schultke S. (2007). The calcium sensor CBL10 mediates salt tolerance by regulating ion homeostasis in Arabidopsis. *Plant J.* 52 473–484. 10.1111/j.1365-313X.2007.03249.x 17825054

[B17] Lee S. C. Lan W. Buchanan B. B. Luan S. (2009). A protein kinase-phosphatase pair interacts with an ion channel to regulate ABA signaling in plant guard cells. *Proc. Natl. Acad. Sci. U.S.A.* 106 21419–21424. 10.1073/pnas.0910601106 19955427 PMC2795491

[B18] Levin J. Z. Horvitz H. R. (1992). The *Caenorhabditis elegans* unc-93 gene encodes a putative transmembrane protein that regulates muscle contraction. *J. Cell Biol.* 117 143–155. 10.1083/jcb.117.1.143 1313436 PMC2289394

[B19] Li J. Wu W. H. Wang Y. (2017). Potassium channel AKT1 is involved in the auxin-mediated root growth inhibition in *Arabidopsis* response to low K^+^ stress. *J. Integr. Plant Biol.* 59 895–909. 10.1111/jipb.12575 28782920

[B20] Luo Q. Wei Q. Wang R. Zhang Y. Zhang F. He Y. (2017). BdCIPK31, a calcineurin b-like protein-interacting protein kinase, regulates plant response to drought and salt stress. *Front. Plant Sci.* 8:1184. 10.3389/fpls.2017.01184 28736568 PMC5500663

[B21] Ma Y. Szostkiewicz I. Korte A. Moes D. Yang Y. Christmann A. (2009). Regulators of PP2C phosphatase activity function as abscisic acid sensors. *Science* 324 1064–1068. 10.1126/science.1172408 19407143

[B22] Marchler-Bauer A. (2015). CDD: NCBI’s conserved domain database. *Nucleic Acids Res.* 43 D222–D226. 10.1093/nar/gku1221 25414356 PMC4383992

[B23] Nguyen T. H. Huang S. Meynard D. Chaine C. Michel R. Roelfsema M. R. G. (2017). A dual role for the OsK5.2 ion channel in stomatal movements and K^+^ loading into xylem sap. *Plant Physiol.* 174 2409–2418. 10.1104/pp.17.00691 28626008 PMC5543972

[B24] Osakabe Y. Arinaga N. Umezawa T. Katsura S. Nagamachi K. Tanaka H. (2013). Osmotic stress responses and plant growth controlled by potassium transporters in *Arabidopsis*. *Plant Cell* 25 609–624. 10.1105/tpc.112.105700 23396830 PMC3608781

[B25] Osakabe Y. Yamaguchi-Shinozaki K. Shinozaki K. Tran L. S. (2014). ABA control of plant macroelement membrane transport systems in response to water deficit and high salinity. *New Phytol.* 202 35–49. 10.1111/nph.12613 24283512

[B26] Park S. Y. Fung P. Nishimura N. Jensen D. R. Fujii H. Zhao Y. (2009). Abscisic acid inhibits type 2C protein phosphatases via the PYR/PYL family of START proteins. *Science* 324 1068–1071. 10.1126/science.1173041 19407142 PMC2827199

[B27] Qiu Q. S. Guo Y. Dietrich M. A. Schumaker K. S. Zhu J. K. (2002). Regulation of SOS1, a plasma membrane Na^+^/H^+^ exchanger in *Arabidopsis thaliana*, by SOS2 and SOS3. *Proc. Natl. Acad. Sci. U.S.A.* 99 8436–8441. 10.1073/pnas.122224699 12034882 PMC123085

[B28] Quan R. Lin H. Mendoza I. Zhang Y. Cao W. Yang Y. (2007). SCABP8/CBL10, a putative calcium sensor, interacts with the protein kinase SOS2 to protect *Arabidopsis* shoots from salt stress. *Plant Cell* 19 1415–1431. 10.1105/tpc.106.042291 17449811 PMC1913747

[B29] Remy E. Cabrito T. R. Batista R. A. Teixeira M. C. Sa-Correia I. Duque P. (2015). The major facilitator superfamily transporter ZIFL2 modulates cesium and potassium homeostasis in Arabidopsis. *Plant Cell Physiol.* 56 148–162. 10.1093/pcp/pcu157 25378686

[B30] Ren X. L. Qi G. N. Feng H. Q. Zhao S. Zhao S. S. Wang Y. (2013). Calcineurin B-like protein CBL10 directly interacts with AKT1 and modulates K^+^ homeostasis in Arabidopsis. *Plant J.* 74 258–266. 10.1111/tpj.12123 23331977

[B31] Shi H. Liu W. Yao Y. Wei Y. Chan Z. (2017). *Alcohol dehydrogenase 1 (ADH1)* confers both abiotic and biotic stress resistance in *Arabidopsis*. *Plant Sci.* 262 24–31. 10.1016/j.plantsci.2017.05.013 28716417

[B32] Silva E. N. Silveira J. A. Rodrigues C. R. Viegas R. A. (2015). Physiological adjustment to salt stress in *Jatropha curcas* is associated with accumulation of salt ions, transport and selectivity of K+, osmotic adjustment and K^+^/Na^+^ homeostasis. *Plant Biol.* 17 1023–1029. 10.1111/plb.12337 25865670

[B33] Spalding E. P. Hirsch R. E. Lewis D. R. Qi Z. Sussman M. R. Lewis B. D. (1999). Potassium uptake supporting plant growth in the absence of AKT1 channel activity: Inhibition by ammonium and stimulation by sodium. *J. Gen. Physiol.* 113 909–918. 10.1085/jgp.113.6.909 10352038 PMC2225604

[B34] Tsay Y. F. Ho C. H. Chen H. Y. Lin S. H. (2011). Integration of nitrogen and potassium signaling. *Annu. Rev. Plant Biol.* 62 207–226. 10.1146/annurev-arplant-042110-103837 21495843

[B35] Wang Y. Wu W. H. (2013). Potassium transport and signaling in higher plants. *Annu. Rev. Plant Biol.* 64 451–476. 10.1146/annurev-arplant-050312-120153 23330792

[B36] Wang Y. Wu W. H. (2017). Regulation of potassium transport and signaling in plants. *Curr. Opin. Plant Biol.* 39 123–128. 10.1016/j.pbi.2017.06.006 28710919

[B37] Weiner J. J. Peterson F. C. Volkman B. F. Cutler S. R. (2010). Structural and functional insights into core ABA signaling. *Curr. Opin. Plant Biol.* 13 495–502. 10.1016/j.pbi.2010.09.007 20934900 PMC2971662

[B38] Xiang J. Ran J. Zou J. Zhou X. Liu A. Zhang X. (2013). Heat shock factor OsHsfB2b negatively regulates drought and salt tolerance in rice. *Plant Cell Rep.* 32 1795–1806. 10.1007/s00299-013-1492-4 23949687

[B39] Xu J. Li H. D. Chen L. Q. Wang Y. Liu L. L. He L. (2006). A protein kinase, interacting with two calcineurin B-like proteins, regulates K^+^ transporter AKT1 in *Arabidopsis*. *Cell* 125 1347–1360. 10.1016/j.cell.2006.06.011 16814720

[B40] Yamaguchi-Shinozaki K. Shinozaki K. (2006). Transcriptional regulatory networks in cellular responses and tolerance to dehydration and cold stresses. *Annu. Rev. Plant Biol.* 57 781–803. 10.1146/annurev.arplant.57.032905.105444 16669782

[B41] Yang T. Zhang L. Hao H. Zhang P. Zhu H. Cheng W. (2015). Nuclear-localized AtHSPR links abscisic acid-dependent salt tolerance and antioxidant defense in Arabidopsis. *Plant J.* 84 1274–1294. 10.1111/tpj.13080 26603028

[B42] Zhu J. K. (2003). Regulation of ion homeostasis under salt stress. *Curr. Opin. Plant Biol.* 6 441–445. 10.1016/S1369-5266(03)00085-2 12972044

[B43] Zong W. Tang N. Yang J. Peng L. Ma S. Xu Y. (2016). Feedback regulation of ABA signaling and biosynthesis by a bZIP transcription factor targets drought-resistance-related genes. *Plant Physiol.* 171 2810–2825. 10.1104/pp.16.00469 27325665 PMC4972276

[B44] Zonia L. Munnik T. (2007). Life under pressure: hydrostatic pressure in cell growth and function. *Trends Plant Sci.* 12 90–97. 10.1016/j.tplants.2007.01.006 17293155

