# Supplementary material for: The Arabidopsis AtUNC-93 Acts as a Positive Regulator of Abiotic Stress Tolerance and Plant Growth via Modulation of ABA Signaling and K+ Homeostasis
Source: Front Plant Sci. 2018 May 30;9:718. doi: 10.3389/fpls.2018.00718 (PMC5989354; doi:10.3389/fpls.2018.00718)
Supplement: Supplementary file 1 [file Table_1.DOC]

**TABLE S1.** Primer sequences used in this study.

| Gene | Primer Name | Primer Sequence (5’-3’) |
| --- | --- | --- |
| For mutants identification | | |
| *AtUNC-93* | *AtUNC-93*-LP | GTTCGGTAATAGGTGTCTT |
|  | *AtUNC-93*-RP | TGTACGGGCTTAGGAAGA |
|  | LBb1.3 | ATTTTGCCGATTTCGGAAC |
|  | LB1 | GCCTTTTCAGAAATGGATAAATAGCCTTGCTTCC |
| For RT-PCR | | |
| *AtUNC-93* | *AtUNC-93*-F | GTTCGGTAATAGGTGTCTT |
|  | *AtUNC-93*-R | TGTACGGGCTTAGGAAGA |
| *ACTIN2* | *ACTIN2*-F | GCAAGTCATCACGATTGGTGC |
|  | *ACTIN2*-R | GCAACGACCTTAATCTTCATGCTG |
| For cloning | | |
| *ProAtUNC-93* | Pro*UNC-93* F | TTCGAGCTCGACGGGTGATTCCTTGG |
|  | Pro*UNC-93* R | AACTGCAGGGCGGAGATTTAGTCCG |
| *AtUNC-93* | *AtUNC-93 GFP* F | CCGCTCGAGCCGGACTAAATCTCCGCC |
|  | *AtUNC-93 GFP* R | GGACTAGTGCTGCGCTCTGCCATACT |
| *AtUNC-93* | *AtUNC-93* OF | CGGGATCCGTGACCGGACTAAATCTC |
|  | *AtUNC-93* OR | AACTGCAGAGTTGCATACTTCCCAAG |
| For real-time PCR | | |
| *AtUNC-93* | *AtUNC-93*-F | TGCTCATAGTGATGCTGGTTATG |
|  | *AtUNC-93*-R | TCAAAGAGTTATTCGTCGTGGG |
| *RD29A* | *RD29A*-F | TCTGACGGCGGTTTAGGAGC |
|  | *RD29A*-R | GTCAAATCCCGTCGGCACAT |
| *RD22* | *RD22*-F | GGAAGAAGCGGAGATGATGAA |
|  | *RD22*-R | GGAAACAGCCCTGACGTGATA |
| *DREB2A* | *DREB2A*-F | TGTAGTTTCAGAGGAGTTAGGCAAAG |
|  | *DREB2A*-R | GCAGCTTCTTGAGCAGTAGGGA |
| *COR15A* | *COR15A*-F | TCAGTTCGTCGTCGTTTCTCAA |
|  | *COR15A*-R | CACCATCTGCTAATGCCTCTTT |
| *COR47* | *COR47*-F | AATCACCAGCGACGACAACA |
|  | *COR47*-R | CGCAGCTAACTCCGGTTCAG |
| *PYR1* | *PYR1*-F | GAACACATCAACGGAAAGACTCG |
|  | *PYR1*-R | AACGGATTTGTAATTCGTCAGCC |
| *PYL2* | *PYL2*-F | CGATGAAGAGCAGAAAACCC |
|  | *PYL2*-R | TAAGAGGCCAAACCACGGAG |
| *PYL4* | *PYL4*-F | TGATCGCGTCGTTTCAAAAAC |
|  | *PYL4*-R | GGCGGAGCAACACTGATTAGG |
| *RCAR1* | *RCAR1*-F | ATGATGGACGGCGTTGAAGG |
|  | *RCAR1*-R | AGAGCAGAGGTACACTGGTTTTCTC |
| *RCAR2* | *RCAR2*-F | AGATGTACGGAGCTCTAGTGACGG |
|  | *RCAR2*-R | CACCAGTGACCAAACAAGATGAAC |
| *RAB18* | *RAB18*-F | CGGGACTGAAGGCTTTGGAAC |
|  | *RAB18*-R | TCATCCTCCGAGCTAGAGCTG |
| *ABI1* | *ABI1*-F | TCCTCAATCCGCCGCTCATTT |
|  | *ABI1*-R | TCTCCTCCGCCAAAGCCAAAT |
| *ABI2* | *ABI2*-F | CATTCAGACCATTCACTGACCCTC |
|  | *ABI2*-R | AGCTCCGTCGCCAGAACAAG |
| *ABI4* | *ABI4*-F | GCGTTAGGGCAGGAACAAGGA |
|  | *ABI4*-R | CCAACGGCGGTGGATGAGTTA |
| *ABI5* | *ABI5*-F | CACATTCTGTTCCGGCTAATCAC |
|  | *ABI5*-R | ACTAGACTCGTTCGCTATCCCTC |
| *SnRK2.2* | *SnRK2.2*-F | CAGTTTCAAGAGCCTGAGCAGC |
|  | *SnRK2.2*-R | AATCATCGAGGCAACGATTACG |
| *SnRK2.3* | *SnRK2.3*-F | CCAGTTCCAGGAGCCTGAACAA |
|  | *SnRK2.3*-R | AATCGTCTAGGCAACGGTTTCG |
| *SnRK2.6* | *SnRK2.6*-F | AGGCACTCAGAATCTGAACCATTA |
|  | *SnRK2.6*-R | CTGTCGATGTCAAGATCATCAAGG |
| *ABF4* | *ABF4*-F | GAGTAGTAACCAGATGAAGCCAACG |
|  | *ABF4*-R | TTTTCCCGGTCCACCTAGTGT |
| *DREB1A* | *DREB1A*-F | CTGCGTTGGCGTTTCAGGATG |
|  | *DREB1A*-R | TTTCGCTCTGTTCCGCCGTGT |
| *MYB2* | *MYB2*-F | AGCCAGAATCATCATCAGCAAT |
|  | *MYB2*-R | ATCATAACCTGACCCGTTCACC |
| *GTG1* | *GTG1*-F | TCGTAATAGTGGTGTAAGAAGGGAG |
|  | *GTG1*-R | CTGCTGAAGGCATAGGGAAAT |
| *GTG2* | *GTG2*-F | ACTTTCTTGGCTATGCGTGTTC |
|  | *GTG2*-R | ATCATCGTGACAGGATCTTTCG |
| *AKT1* | *AKT1*-F | GAACTGGAAACCCGGTGAG |
|  | *AKT1*-R | AACCCAATTCTAGCAACTCCTT |
| *AKT2* | *AKT2*-F | AGTTGGAGCACTTTGTTG |
|  | *AKT2*-R | CTTGAGCATTGTGGCATT |
| *AKT5* | *AKT5*-F | CGCACCATTAATACTTCC |
|  | *AKT5*-R | CACCATCTTCTTCCTCTT |
| *AKT6* | *AKT6*-F | AACTATTTGTCTTAACGCAA |
|  | *AKT6*-R | ATATTTCAATCCCGGAGT |
| *GORK* | *GORK*-F | CGTCTATCTACCCGTCAA |
|  | *GORK*-R | AACTCCGTGCTTTCTACT |
| *SKOR* | *SKOR*-F | TCCAAAGAGCCTAAAGAT |
|  | *SKOR*-R | TCGAGGTACCCACAGTAC |
| *KAT1* | *KAT1*-F | AATCCGACTTCCGACACT |
|  | *KAT1*-R | TCTTTCCACTTTGGCTCT |
| *KAT2* | *KAT2*-F | TCAGAACCAGAGTCACTT |
|  | *KAT2*-R | GAATCATGCAGTTGTAAA |
| *KAT3* | *KAT3*-F | ATCTGGAGGAGTGGATAT |
|  | *KAT3*-R | GTGAAAGGCTGAGGAATG |
| *KCO1* | *KCO1*-F | CTAATAGTTCCGCCTCAA |
|  | *KCO1*-R | AAGCCTCTTGTTTCTCCACT |
| *KCO2* | *KCO2*-F | TTCATTCGTTCGTTCTAG |
|  | *KCO2*-R | GGTTTATTAGGCTCCGTA |
| *KCO3* | *KCO3*-F | TTATCAACAACAAGGACCCA |
|  | *KCO3*-R | TGACCGAGGTAATGAGCC |
| *KCO4* | *KCO4*-F | GGATTAGTGAGGATGATA |
|  | *KCO4*-R | TAGTACCAGACAAGAGTTAG |
| *KCO5* | *KCO5*-F | TCGGAAAGCTGTTAAATT |
|  | *KCO5*-R | TGAGTGATCTTCCCCATT |
| *KCO6* | *KCO6*-F | ACGGATCAACGATCCTAA |
|  | *KCO6*-R | GCAAGACAAGACCAAAGA |
| *ACTIN2* | *ACTIN2*-F | GTTTGTGGGAATGGAAGC |
|  | *ACTIN2*-R | CTCATACGGTCAGCGATA |
