# Supplementary material for: The Arabidopsis AtUNC-93 Acts as a Positive Regulator of Abiotic Stress Tolerance and Plant Growth via Modulation of ABA Signaling and K+ Homeostasis
Source: Front Plant Sci. 2018 May 30;9:718. doi: 10.3389/fpls.2018.00718 (PMC5989354; doi:10.3389/fpls.2018.00718)
Supplement: Supplementary file 2 [file Data_Sheet_1.doc]

**Supplementary Figure Legends**

**FIGURE S1.** Multiple sequence alignment of UNC-93 orthologs from different plant species. Sequences in the alignment include AT3G09470 from *Arabidopsis thaliana*, BnaC05g43430D from *Brassica napus*, XP_006480787 from *Citrus sinensis*, XP_002273762 from *Vitis vinifera*, XP_016669733 from *Gossypium hirsutum*, Gm19g173000 from *Glycine max*, BAJ98265 from *Hordeum vulgare*, Os12g0566900 from *Oryza sativa*, Zm2g007146 from *Zea mays* and Sb08g018540 from *Sorghum bicolor*. Dark blue indicates 100% identity of amino acid sequences. Pink indicates identity less than 100% and greater than or equal to 75%. Light blue indicates identity less than 75% and greater than or equal to 50%. Yellow indicates identity less than 50% and greater than or equal to 33%. UNC-93 indicates UNC-93 domain.

**FIGURE S2.** Phylogenetic analysis of UNC-93 orthologs from different species. An unrooted consensus phylogenetic tree was constructed with 18 UNC-93 orthologs from different species. The species and protein names or number were given in the phylogenetic tree. Tree reconstruction using the neighbor-joining method and confirmation of tree topology by bootstrap analysis (1,000 replicates) were performed with MEGA5.0 software.

**FIGURE S3.** Phenotype tests of wild type, *atunc-93* mutants and *AtUNC-93*-overexpressing seedlings under salt and osmotic stresses. **(A,B)** Four-day-old seedlings were transferred to 1/2 MS medium with different concentrations of NaCl **(A)** or mannitol **(B)**. Photographs were taken after 14 d NaCl or 20 d mannitol treatment. **(C,D)** The survival rates of various plant materials after 14 d NaCl **(C)** or 20 d mannitol **(D)** treatment were determined as the number of visible green plants. Data are shown as means ± SE (n = 3). Student’s *t* test (*P < 0.05 and **P < 0.01) was used to analyze statistical significance compared with the wild type.

**FIGURE S4.** Phenotype tests of wild type, *atunc-93* mutants and complementation lines under salt and osmotic stresses. **(A)** RT-PCR verification of *AtUNC-93* expression in the complementation lines. **(B,C)** Phenotype comparison of 4-day-old seedlings after treatments with NaCl or mannitol for 10 d. **(D,E)** The survival rates of various plant materials as indicated after 10 d NaCl **(D)** or mannitol **(E)** treatment were determined as the number of visible green plants. Data are shown as means ± SE (n = 3). Student’s *t* test (*P < 0.05 and **P < 0.01) was used to analyze statistical significance compared with the wild type.

**FIGURE S5.** Phenotype tests of wild type, *atunc-93* mutants and *AtUNC-93*-overexpressing seedlings under temperature stress. **(A)** Phenotype of 4-day-old seedlings under heat stress as temperature-increasing programmes on the right. Photographs were taken after recovery at 22 ^o^C for 7 d. **(B)** Phenotype of 4-day-old seedlings under cold stress treatment at 4 ^o^C for 10 d and recovered at 22 ^o^C for 7 d. Seedlings cultured under normal growth conditions were used as controls. **(C)** Schematic position of wild type (WT), *atunc-93* mutants (*atunc-93-1* and *atunc-93-2*) and *AtUNC-93*-overexpressing (OE1) seedlings under heat **(A)** and cold **(B)** stresses.

**FIGURE S6.** Expression of ABA-responsive genes in *atunc-93* mutants and *AtUNC-93*-overexpressing lines. The expression levels of a set of ABA-responsive genes were tested by using real-time PCR under the treatments with or without ABA. Each data bar represents the mean ± SE (n = 3).

**FIGURE S7.** Phenotype of the wild type, *atunc-93* mutants and *AtUNC-93*-overexpressing lines. **(A)** Seedlings grown on 1/2 MS medium in the light for 7 d. Bars = 10 mm. **(B)** Seedlings grown on 1/2 MS medium in the dark for 10 d. Bars = 10 mm. **(C)** Root or hypocotyl lengths of various plant materials grown in the light for 7 d or in the dark for 10 d as described in **(A)** and **(B)**. **(D)** Phenotype comparison of 14-day-old various plant materials grown on 1/2 MS medium in the light. Bars = 5 mm. **(E,F)** Dry weight of the roots **(E)** and shoots **(F)** of 14-day-old seedlings grown on 1/2 MS medium as described in **(D)**. Data in **(C,E,F)** are shown as means ± SE (n = 3). Student’s *t* test (*P < 0.05) was used to analyze statistical significance compared with the wild type.

**FIGURE S8.** Phenotype tests of the wild type, *atunc-93* mutants and *AtUNC-93*-overexpressing seedlings under low-K^+^ conditions. **(A)** Phenotype comparison of 4-day-old seedlings after being transferred to medium containing different concentrations of K^+^ for 10 d. Seedlings cultured on MS medium were used as controls. **(B)** Expression of K^+^ channel genes in various plant materials was analyzed by using real-time PCR. Data represent the means ± SE (n = 3).

**FIGURE S9.** Na^+^ content measurements of the wild type, *atunc-93* mutants and *AtUNC-93*-overexpressing lines. **(A,B)** Na^+^ content **(A)** and K^+^/ Na^+^ ratio **(B)** of various plant materials as indicated under MS and LK conditions. Data are shown as means ± SE (n = 3). Student’s *t* test (*P < 0.05) was used to analyze statistical significance compared with the wild type.
