# Supplementary figures and images for: The Arabidopsis AtUNC-93 Acts as a Positive Regulator of Abiotic Stress Tolerance and Plant Growth via Modulation of ABA Signaling and K+ Homeostasis
Source: Front Plant Sci. 2018 May 30;9:718. doi: 10.3389/fpls.2018.00718 (PMC5989354; doi:10.3389/fpls.2018.00718)

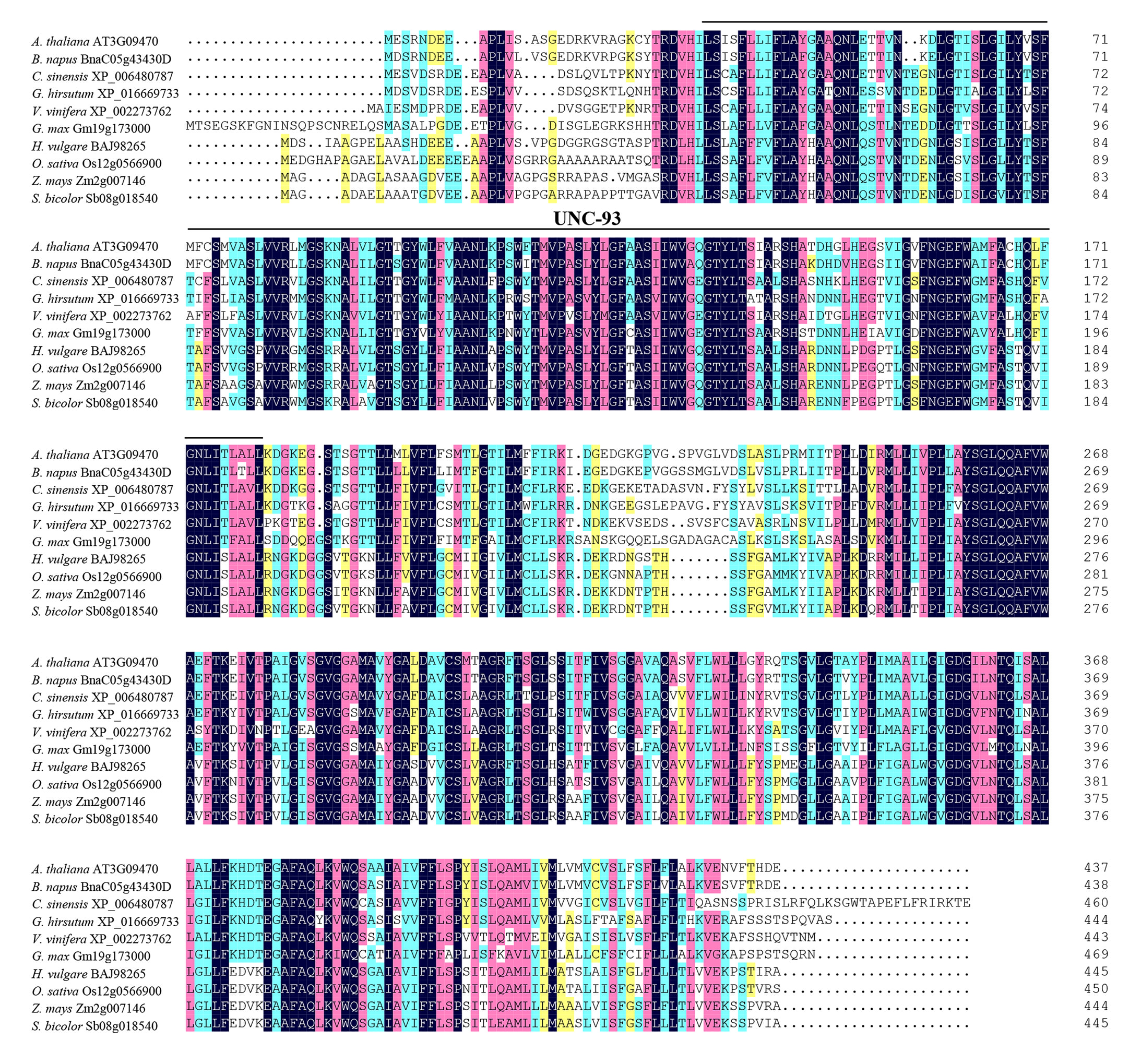

Supplement: Supplementary file 3 [file Image_1.TIF]

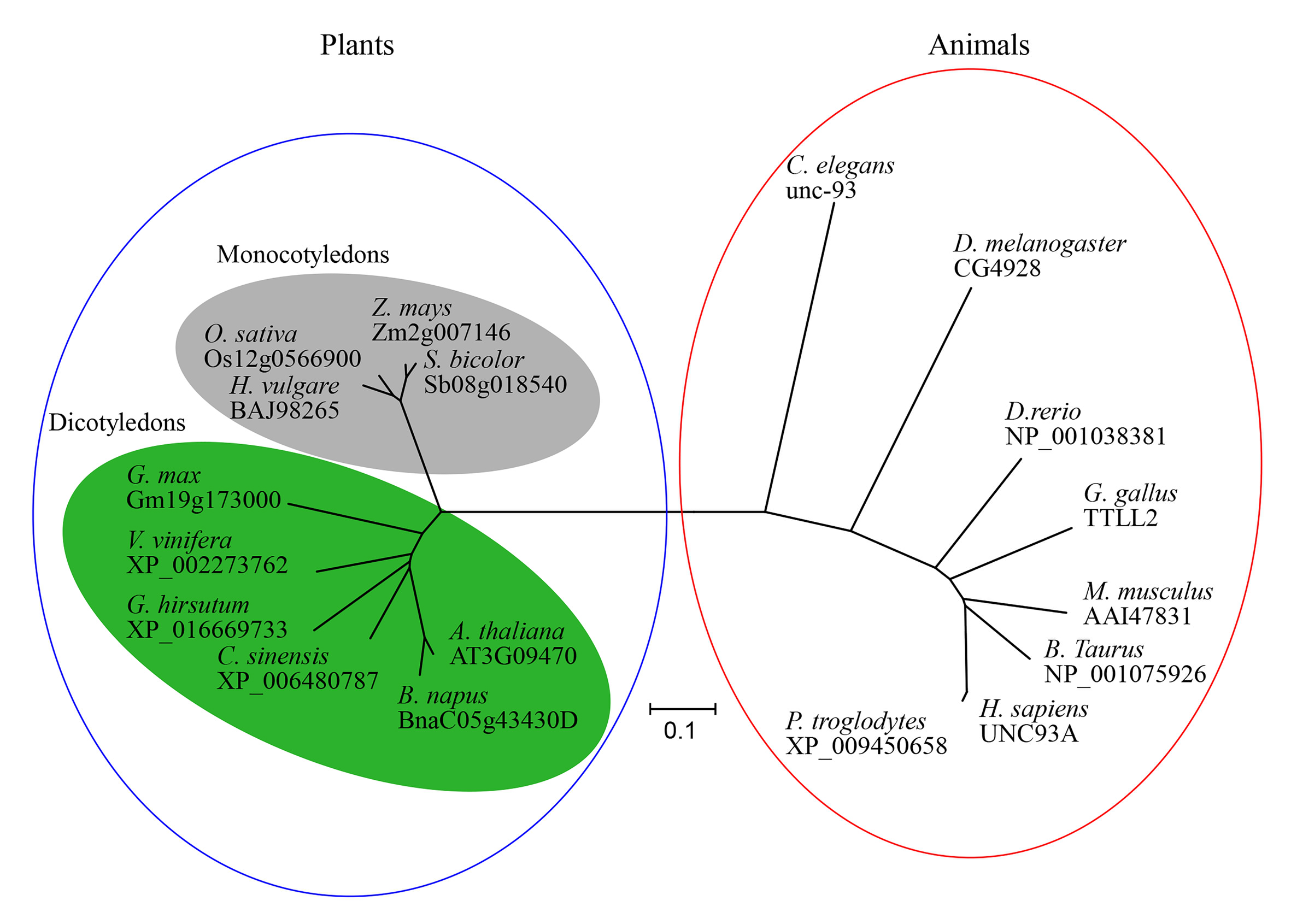

Supplement: Supplementary file 4 [file Image_2.TIF]

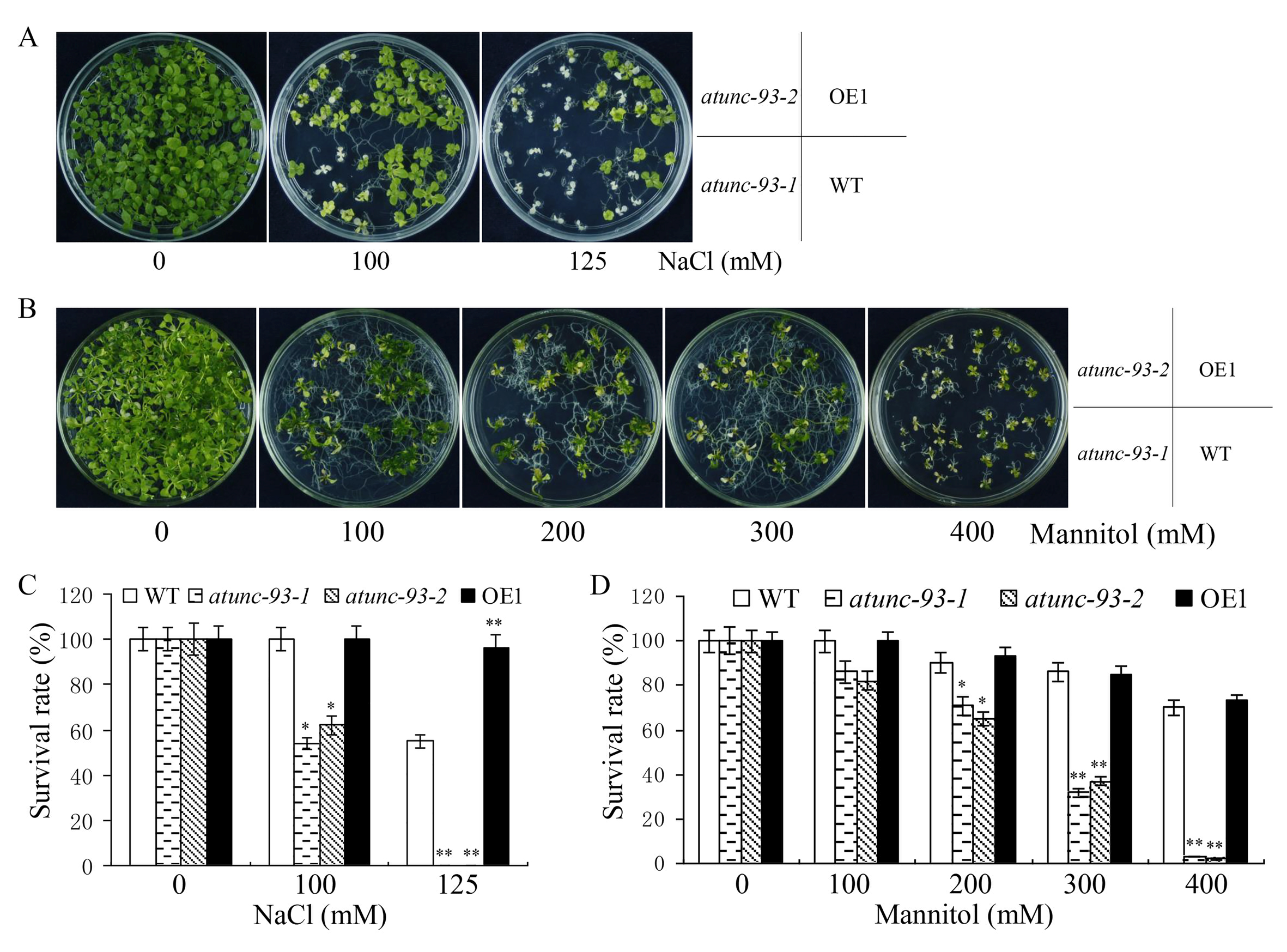

Supplement: Supplementary file 5 [file Image_3.TIF]

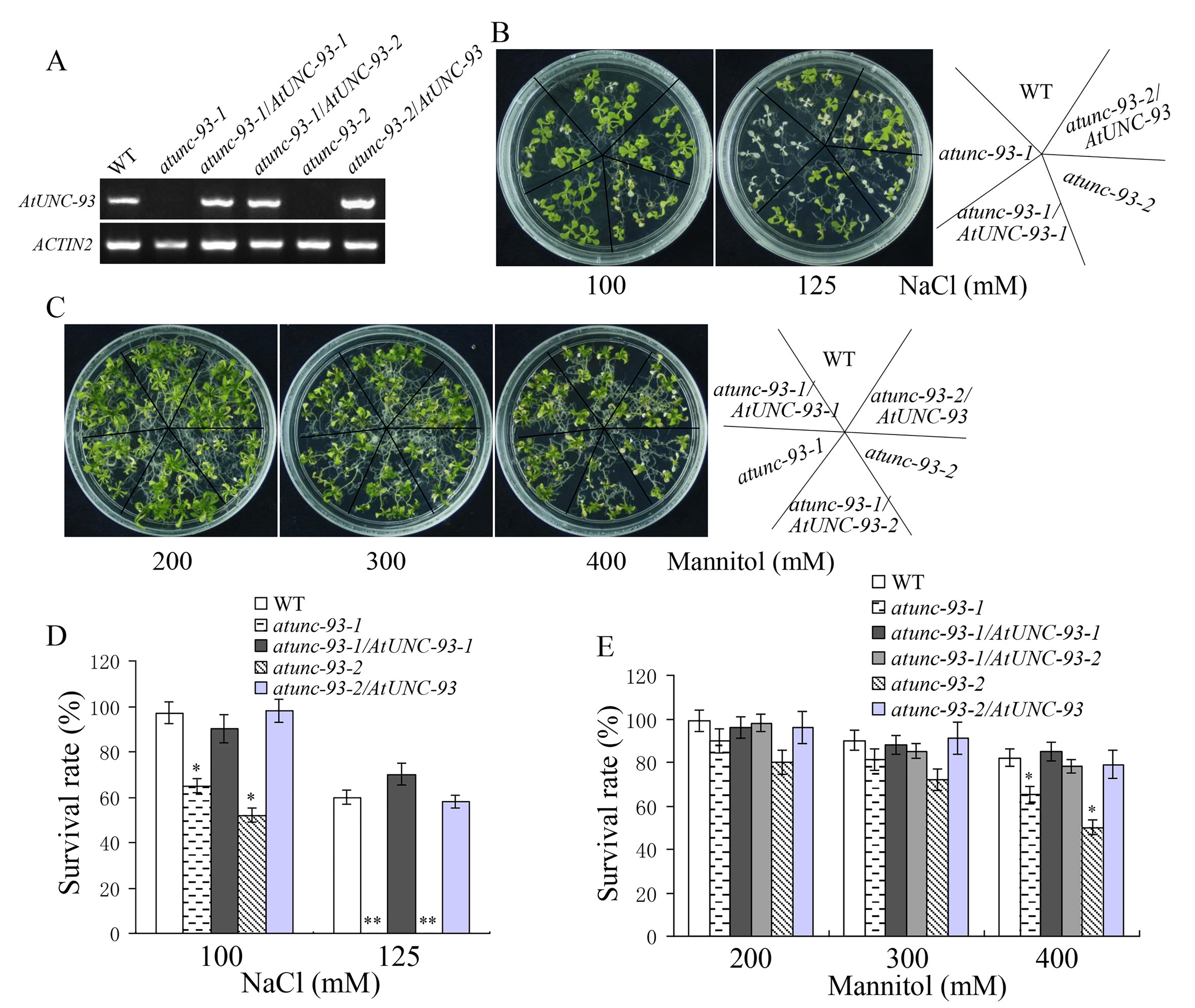

Supplement: Supplementary file 6 [file Image_4.TIF]

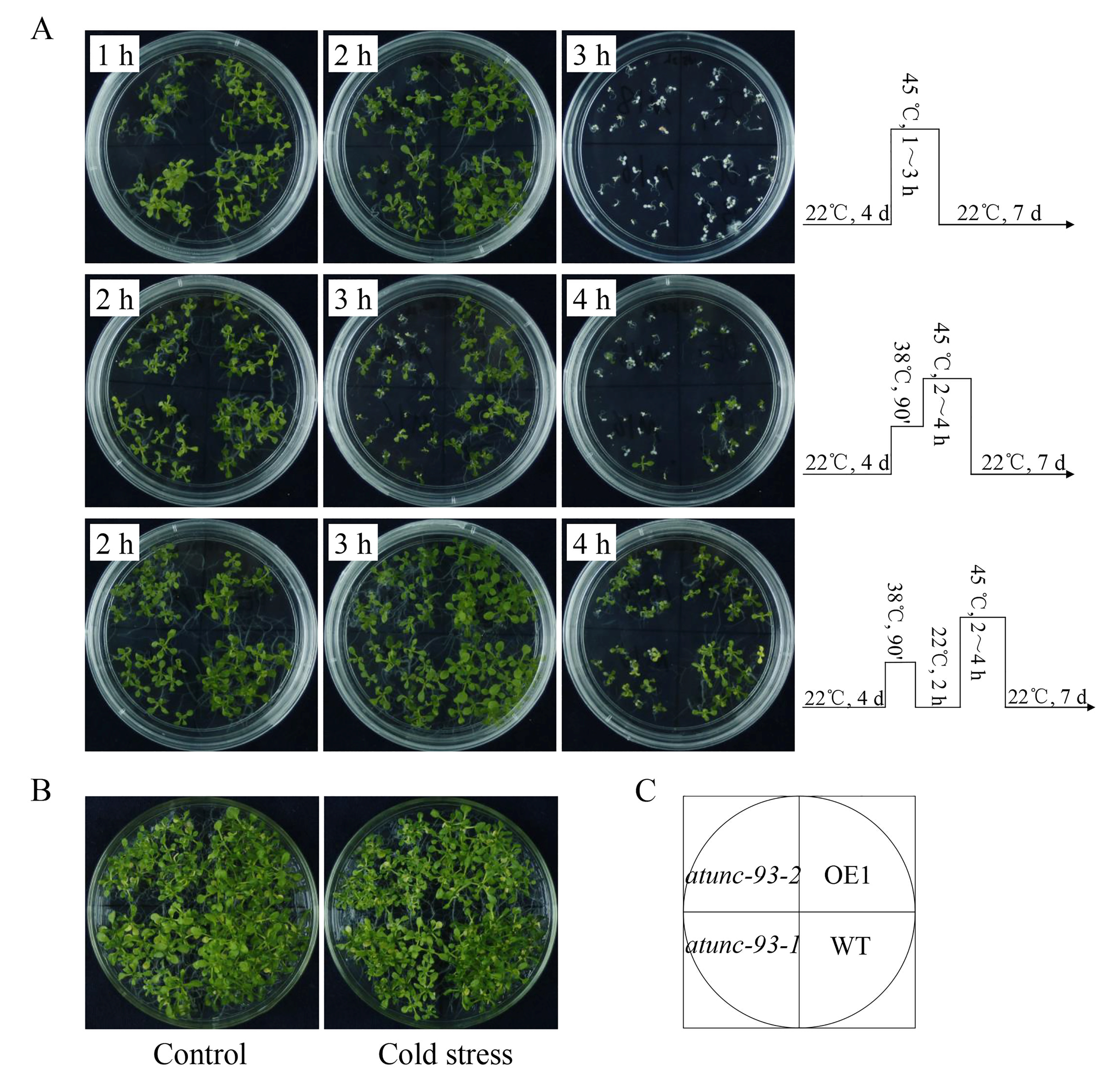

Supplement: Supplementary file 7 [file Image_5.TIF]

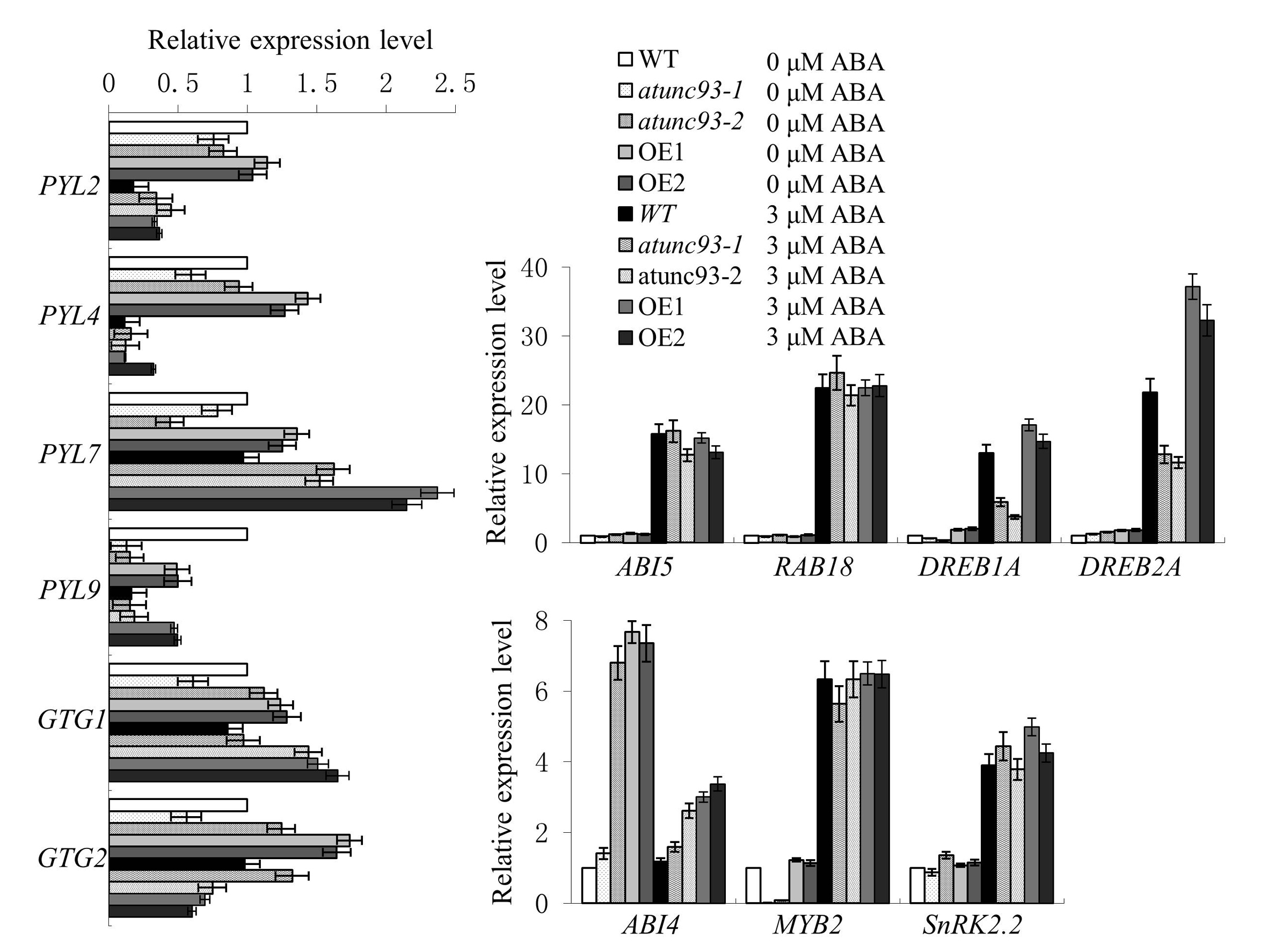

Supplement: Supplementary file 8 [file Image_6.TIF]

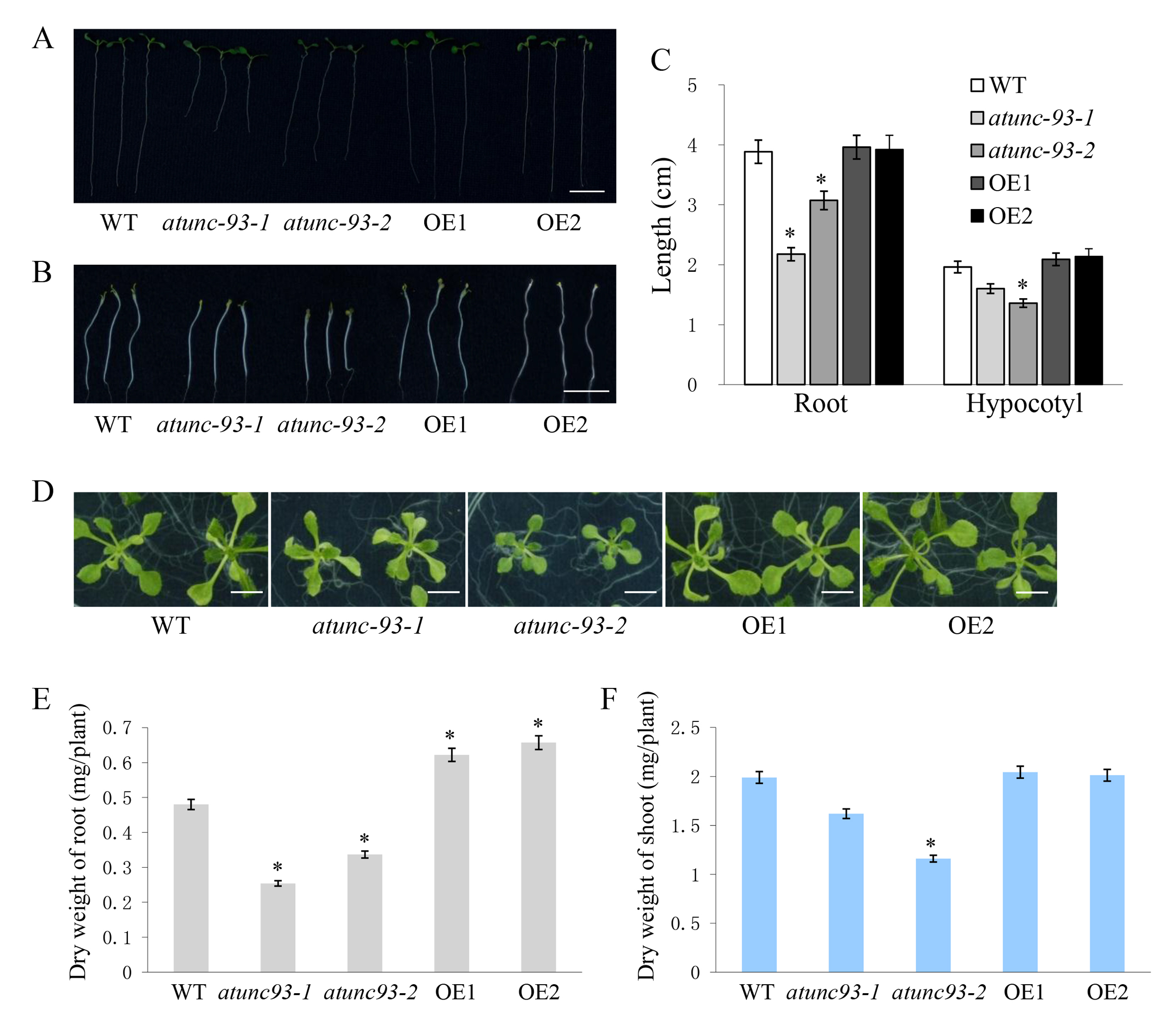

Supplement: Supplementary file 9 [file Image_7.TIF]

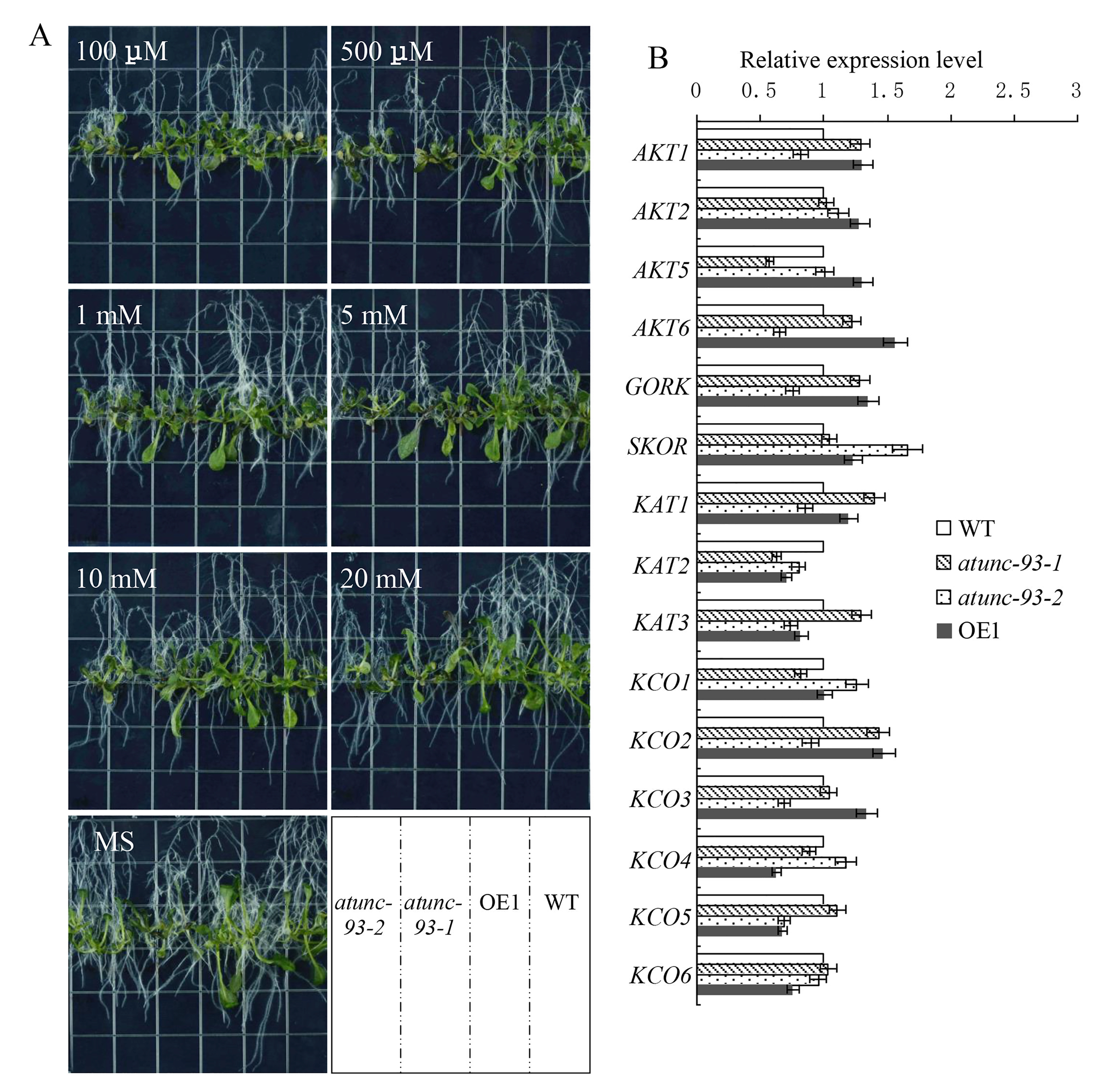

Supplement: Supplementary file 10 [file Image_8.TIF]

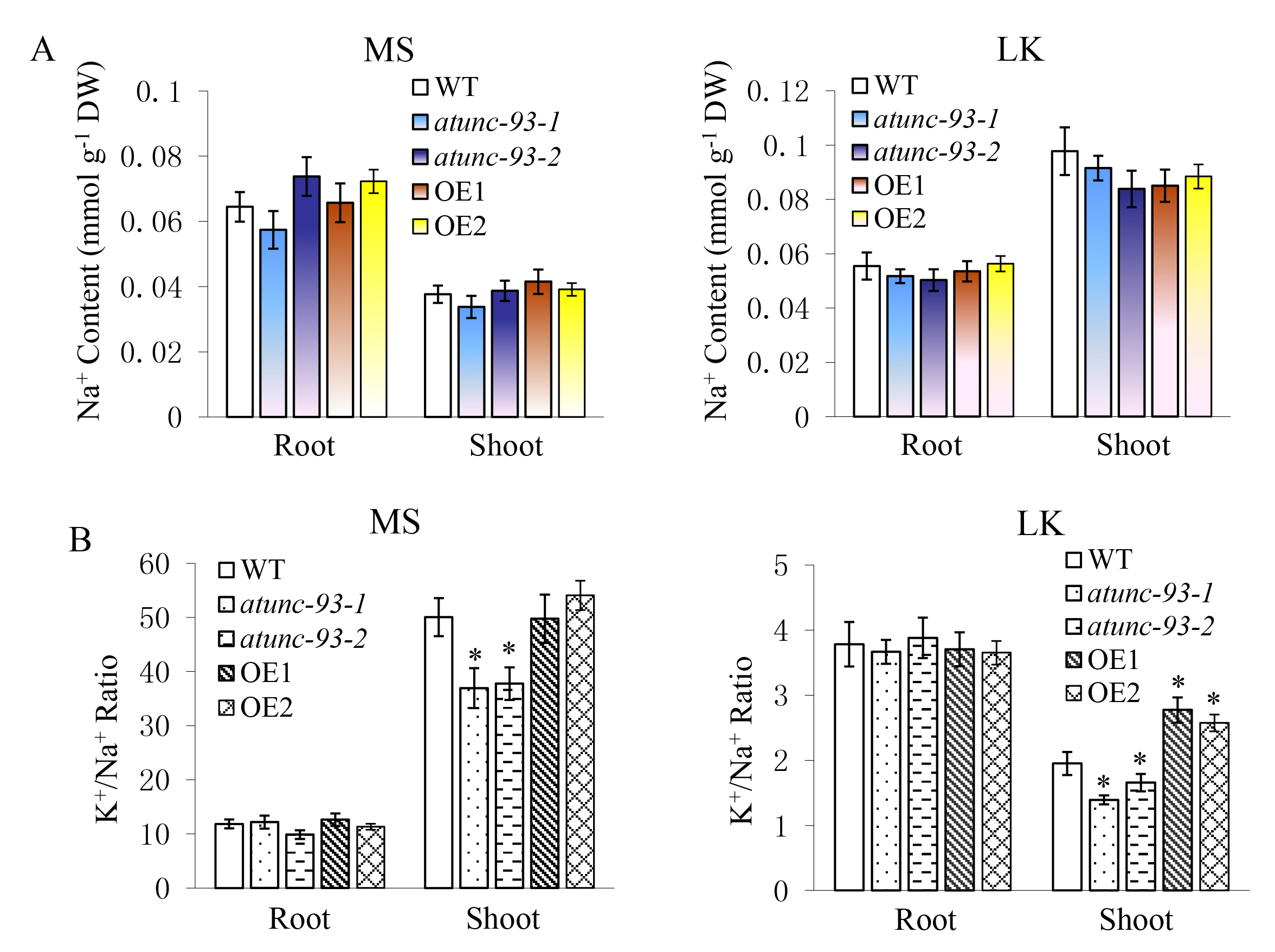

Supplement: Supplementary file 11 [file Image_9.TIF]
